# Arabidopsis Seed Stored mRNAs are Degraded Constantly over Aging Time, as Revealed by New Quantification Methods

**DOI:** 10.3389/fpls.2019.01764

**Published:** 2020-01-29

**Authors:** Liang Zhao, Sheng Wang, Yong-Bi Fu, Hong Wang

**Affiliations:** ^1^ Plant Gene Resources of Canada, Saskatoon Research and Development Centre, Agriculture and Agri-Food Canada, Saskatoon, SK, Canada; ^2^ Department of Biochemistry, Microbiology and Immunology, University of Saskatchewan, Saskatoon, SK, Canada

**Keywords:** *Arabidopsis thaliana*, real-time quantitative PCR (qPCR), seed aging, seed germination, stored mRNA, long-lived mRNA

## Abstract

How plant seeds age remains poorly understood and effective tools for monitoring seed aging are lacking. Dry seeds contain various stored mRNAs which are believed to be required for protein synthesis during early stages of seed germination. We reasoned that seed stored mRNAs would undergo degradation during seed aging, based on the propensity of mRNAs to degrade. We performed RT-PCR and qPCR analyses to study the changes in stored mRNA levels of Arabidopsis seeds during aging. All stored mRNAs analyzed were gradually degraded in both naturally and artificially aged seeds. The difference in Ct values between aged and control seeds (ΔCt value) was highly correlated with the mRNA fragment size and seed aging time. We derived mathematical equations for estimating the relative amount of undamaged stored mRNAs and frequency of the breakdown at one nucleotide level for individual mRNAs. Stored mRNAs were found to break down randomly. The frequency of breaks per nucleotide per day, which we named *β* value, remained fairly constant under the same aging conditions over aging time. This parameter should allow the effects of different conditions on the degradation of stored mRNAs to be quantitatively compared. Also, we showed that the change in stored mRNA levels could serve as a more precise biomarker for seed aging assessment than three existing methods. These methods and findings will advance the studies of stored mRNAs and seed ageing in plants, and likely slow RNA degradation in non-plant systems.

## Introduction

Plant seeds age gradually during storage and eventually lose the ability to germinate (Finch-Savage and Leubner-Metzger, 2006; Holdsworth et al., 2008). Aged seeds tend to have reduced seed vigor and could affect agricultural production (Oge et al., 2008; Rajjou and Debeaujon, 2008; Marcos Filho, 2015). In addition, conserving and regenerating about 7.4 million accessions of seed germplasm held in more than 1,750 genebanks around the world are facing technical challenges (FAO, 2010), as standard germination tests are not always effective (Fu et al., 2017). However, our understanding on how seeds age remains limited and effective tools for monitoring seed aging are lacking (Fu et al., 2015).

It has been suggested that seed longevity of the orthodox seeds which can survive desiccation and freezing is mainly due to the formation of the glassy state (Pereira Lima et al., 2017). In this extremely viscose state, chemical reactions were reduced because of the limited molecular diffusion and low availability of free water (Fernandez-Marin et al., 2013). However, seed deterioration and aging still continues at a slow speed under storage (Kranner et al., 2010). Although the exact causes of seed aging are still not fully understood, reactive oxygen species (ROSs) have been suggested as the major contributor of seed aging (Lehner et al., 2008; Yao et al., 2012). For seeds in storage, ROSs can be produced through non-enzymatic reactions such as Maillard reactions between reduced sugars and molecules with free amino groups (Miyata et al., 2000; Murthy and Sun, 2000) and lipid peroxidation (Held, 2012). The accumulated ROSs can cause damages to macromolecules like nucleic acids (Yang et al., 2001; Nakabayashi et al., 2005; Kimura and Nambara, 2010; Chen et al., 2012), proteins (Prieto-Dapena et al., 2006; Oge et al., 2008; Almoguera et al., 2009) and lipids (Sattler et al., 2004; Levesque-Tremblay et al., 2009; Bueso et al., 2014) as well as organelles (Gadaleta et al., 1990). In this regard, it is interesting to note that overexpression of certain genes involved in scavenging ROSs or repairing damaged macromolecules have been reported to improve seed longevity, including overexpressing in tobacco both *Pisum sativum* Cu/Zn-superoxide dismutase (CuZnSOD) and ascorbate peroxidase (APX) (Gupta et al., 1993; Lee et al., 2010), in Arabidopsis the sacred lotus *NnMT2a* and *NnMT3*, which are members of the metallothioneins involving ROS scavenging (Zhou et al., 2012), in rice a rice heat shock protein OsHSP18.2 (Kaur et al., 2015), and in rice the *Pseudomonas* Aldo-ketoreductase 1 (PsAKR1), which is shown to function as an enzyme in reducing reactive carbonyl compounds (Nisarga et al., 2017). Further, the damage on DNA is also considered as a factor in seed aging (Chen et al., 2012). One common product of DNA damage caused by ROS is 8-oxo-G which can result in the transversion from GC to TA in the subsequent DNA replication (Yoshida et al., 2002). The 8-oxo-G on DNA could be repaired by 8-oxoguanine-DNA glycosylase 1 (OGG1) which functions as glycosylase/ apurinic/ apyrimidinic (AP) lyase to excise the damaged base (Nash et al., 1996). It was shown that overexpressing *OGG1* in Arabidopsis decreased the level of 8-oxo-G and rendered the seeds more resistant to seed aging treatment, indicating that DNA damage contribute to seed deterioration (Chen et al., 2012).

Mature plant seeds contain various mRNAs referred to as stored mRNAs (Marcus and Feeley, 1964; Dure and Waters, 1965), and also long-lived mRNAs because they are present in seeds from late embryogenesis to early seed germination (Sano et al., 2015). In *Arabidopsis* seeds, over 12, 000 stored mRNA species were detected (Nakabayashi et al., 2005) and different varieties have similar stored mRNAs (Kimura and Nambara, 2010). In addition, the classes of long-lived mRNAs are highly conserved between dicot *Arabidopsis* and monocot barley seeds (Rajjou et al., 2012), suggesting functional importance of seed stored mRNAs.

The stored mRNAs encode many proteins of diverse processes (Nakabayashi et al., 2005; Sano et al., 2012; Sano et al., 2015) and may be needed for protein synthesis during early seed germination (Comai et al., 1989; Rajjou et al., 2004; Kimura and Nambara, 2010; Sano et al., 2015). When germinating *Arabidopsis* seeds were treated with α-amanitin, a potent inhibitor of DNA-dependent RNA polymerase II, seeds could still germinate successfully although the seed vigor is severely affected; but they failed to germinate when treated with cycloheximide, a protein translation inhibitor, suggesting that stored mRNAs could support initial seed germination without the synthesis of new mRNAs (Rajjou et al., 2004). Sano et al. (2015) showed that in rice stored mRNAs increased from 10 days after flowering (DAF) and became highly abundant at 40 DAF. While the germination of 10-DAF embryos was severely impaired by the transcriptional inhibitor actinomycin D (Act D), over 70% embryos of 20–40 DAF could germinate in the presence of ActD, supporting that accumulation of stored mRNAs is critical for seed germination and the resistance to ActD inhibition.

There has been little research examining the changes of stored mRNAs during seed aging, although many studies have reported the degradation of ribosomal RNAs. Earlier on, it was observed that the amount and integrity of rRNAs decreased in non-viable embryos of rye grains (Roberts et al., 1973) and pea embryonic axis tissues (Bray and Chow, 1976). Deterioration in rRNA integrity was observed in seeds of plants such as carrot, tobacco, sunflower and soybean, based on the appearance of shorter RNA fragments supposedly due to the degradation of 25S and 18S rRNAs (Brocklehurst and Fraser, 1980; Thompson et al., 1987; Reuzeau and Cavalie, 1997) or on the relative amount of intact 25S and 18S rRNAs (Brocklehurst and Fraser, 1980; Kranner et al., 2011; Fleming et al., 2017).

Since mRNAs are prone to degradation in general, we reasoned that seed stored mRNAs would undergo degradation during seed aging and thus could serve as molecular markers for monitoring seed aging. In this study, using Arabidopsis as a model, we thoroughly examined the relationships among seed aging time, seed germination and stored mRNA levels in naturally aged (NA) and artificially (or accelerated) aged (AA) seeds. We developed new methods for quantifying the changes in stored mRNA levels and the rate of changes at one nucleotide level. Our results show that seed stored mRNAs are degraded at a constant rate under the same aging conditions. Further, the change in one stored mRNA can be used as a good measurement of seed aging.

## Results

### RT-PCR (Reverse Transcription PCR) Analysis of Arabidopsis Seed Stored mRNA Levels during Seed Aging

To study the changes in stored mRNAs during seed aging, we surveyed 120 genes, which were used previously in our other research activities, for the presence of stored mRNAs in Arabidopsis seeds. The genes belong to different gene families and have diverse functions, and were loosely grouped into four groups in protein ubiquitination and degradation (group A), heat shock proteins in protein protection (group B), cell cycle and plant growth (group C) and other functions including ROS scavenging, stress response, ABA signaling and transcriptional regulation (group D) (**Table S2**). For ease of identification, they were referred to by the simple codes such as A5, B10, etc. (**Table S2**). Since these genes belong to many different families and are diverse in their functions, sequences, and lengths, changes in their stored mRNAs during seed aging should reflect the changes occurring to stored mRNAs in general. RT-PCR analysis detected the presence of stored mRNAs for about 83 genes (**Figure S1**), indicating that about 69% of the 120 genes surveyed had a detectable level of stored mRNAs in dry seeds under the present conditions.

**Figure 1:**
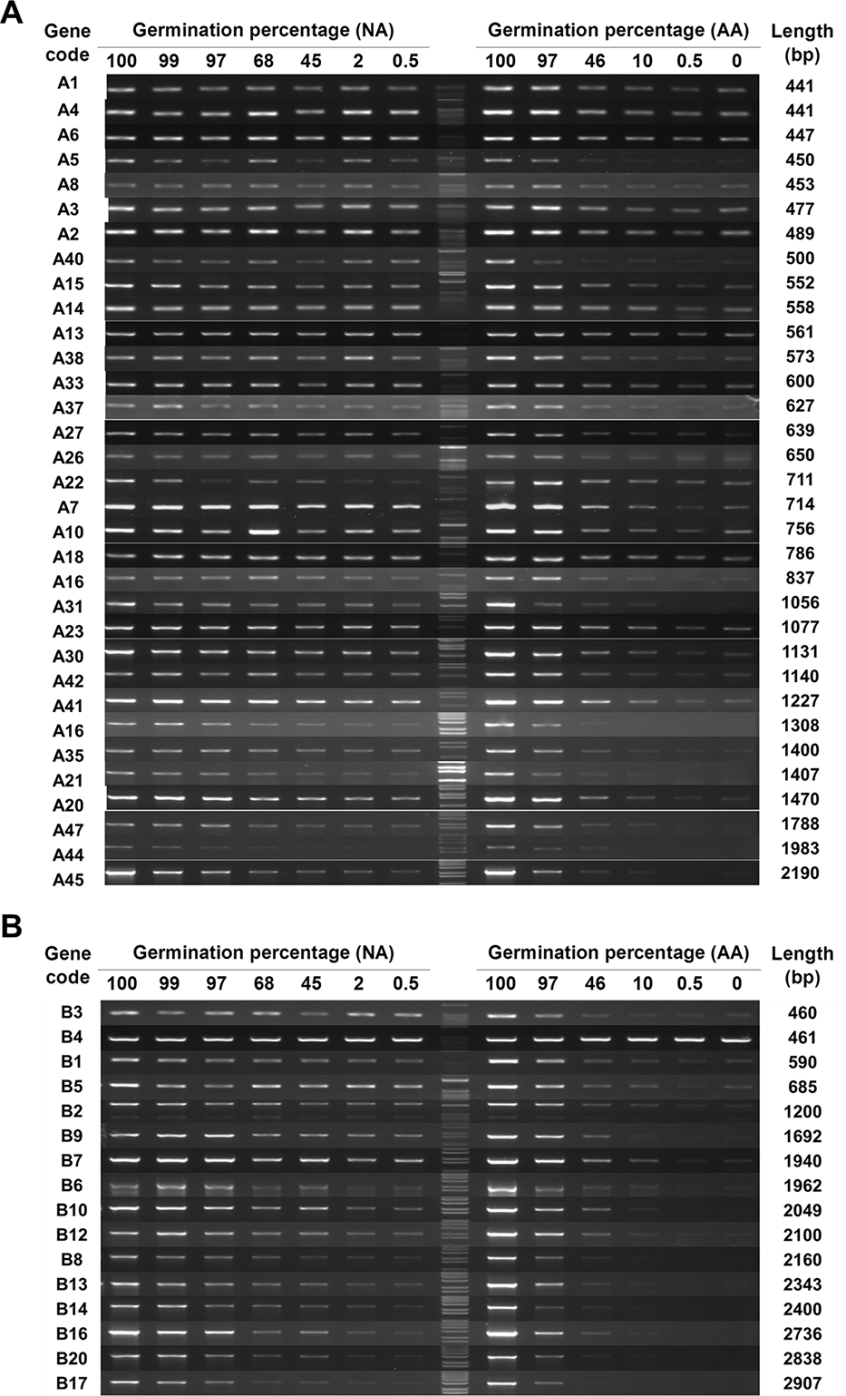
The level of stored mRNAs in naturally aged (NA) and artificially aged (AA) Arabidopsis seeds detected by RT-PCR. cDNAs from NA and AA seeds with different percentages of germination as well as the control seeds (100% germination) were used. Different genes in two groups were analyzed. The number of PCR cycles varied depending on their mRNA abundance in the unaged seeds. PCR products were run in agarose gels. **(A)** Analysis of 33 genes in group A (see **Table S2** for the list of genes). **(B)** Analysis of 16 genes in group B. The germination percentage of a seed sample is indicated above the DNA lane. M stands for the 1 kb DNA molecular ladder. The gene code is indicated at the left and the cDNA length amplified by PCR indicated at the right of each row.

The 83 genes (33, 16, 19, and 15 genes from A, B, C, and D groups, respectively) with a detectable level of stored mRNA were then used to determine the changes in stored mRNAs during seed aging. Since cDNAs were synthesized from stored mRNAs, the damage or lesion occurring to a stored mRNA occurring during seed aging should be reflected by the change in its cDNA level. RT-PCR results showed that almost all stored mRNAs showed a gradual decrease in both NA and AA seed samples, but the extent of decrease varied greatly among different genes (**Figures 1** and **S2**; the original gel images available in **Data Sheet 1**). In examining the data, it became clear that longer cDNA fragments showed a greater decrease in general. For easy viewing, genes in **Figures 1** and **S2** are presented according to the length of cDNA coding region used in the analysis. The trend was more apparent for the genes in group B, with the coding region ranging from 460bp to 2,900bp (bp stands for base pairs) while most of the genes in group A have a length less than 1,500bp. Thus, the stored mRNAs showed a decrease during both natural and artificial seed aging, with longer mRNAs showing a greater decrease. Although RT-PCR is only a “semi-quantitative” technique, the results provide a good overview regarding the changes in stored mRNAs during seed aging.

### qPCR (Real-Time Quantitative PCR) Analysis of Seed Stored mRNA Levels During Seed Aging

**Figure 2:**
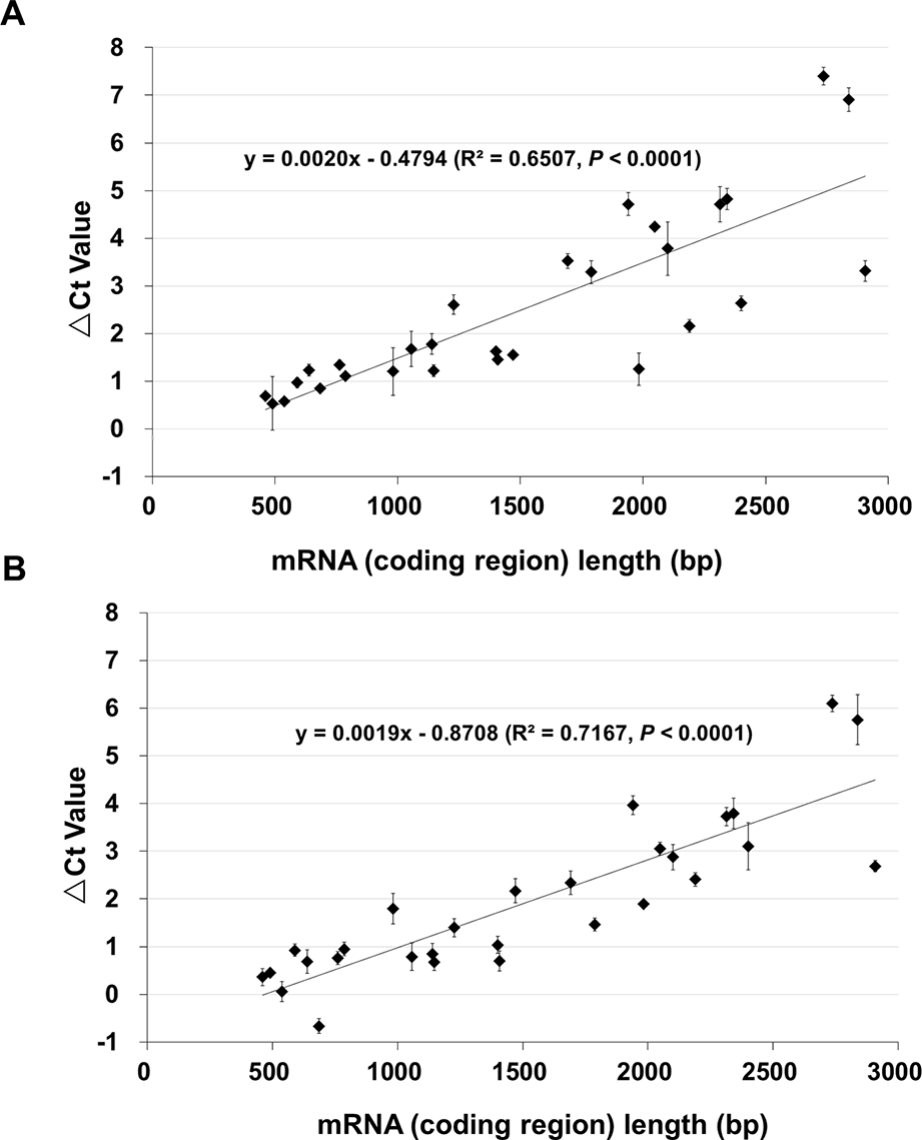
The relationship between the length of stored mRNAs and ΔCt value in qPCR analysis. cDNAs of unaged control, AA (artificially aged, 0.5% germination) and NA (naturally aged, 0.5% germination) seeds were used. qPCR analysis was conducted for 29 genes which differ in the cDNA (coding region) length. For each gene, the difference in the Ct value between the aged and control samples was obtained as ΔCt = Ct of the aged sample – Ct of control. Each ΔCt value was the average of three biological repeats (with the standard error shown). **(A)** ΔCt values of 29 genes between the AA and control seeds, and the correlation with the cDNA length. **(B)** ΔCt values of 29 genes between the NA and control seeds, and the correlation with the cDNA length. The linear regressions were produced with Microsoft Excel 2010 and both have a P < 0.0001.

To quantitatively determine the changes, qPCR was performed using cDNAs of control (100% germination), NA (0.5% germination) and AA (0.5% germination) seeds. For this analysis, we used 29 genes mostly from A and B groups. They had a DNA band of the anticipated size from the RT-PCR analysis and represented various lengths in the coding region from 461 to 2,907 bp (**Table S3**). The changes in Ct values (or ΔCt values) of the 29 genes for AA and NA seeds are shown in **Table S3**. When the ΔCt values were plotted against the cDNA coding region length (**Figure 2**), there was a good correlation between the ΔCt value and fragment length for both AA (**Figure 2A**) and NA (**Figure 2B**) seeds (R^2^ = 0.6507 and P < 0.0001 for AA data, and R2 = 0.7167 and P < 0.0001 for NA data). Remarkably, the slopes of the regression equations for AA and NA seeds were very similar (0.0020 and 0.0019, respectively) (**Figure 2**), indicating a similar linear relation between the ΔCt value and length of stored mRNAs in NA and AA seeds with same percentage of germination. Also, for one gene (B4), the qPCR data showed a decrease in both NA and AA seeds (**Table S3**), which the RT-PCR analysis could not clearly detect. These findings further showed that the levels of all stored mRNAs analyzed decreased in aged seeds and the extent of decrease was correlated with mRNA length.

### Analysis of the Relationship Between mRNA Fragment Length and ΔCt Value Using the Same Genes

The observation that the ΔCt value between aged and control seed samples depended on the cDNA fragment length is interesting. Since for the initial analysis different genes were used, the ΔCt value could potentially be affected by other factors in addition to the cDNA fragment length. To more clearly determine the relationship between mRNA fragment length and the decrease in the mRNA level, we used different fragment lengths of the same stored mRNAs, and designed primers with annealing temperatures about 60°C for better specificity and consistency.

**Figure 3:**
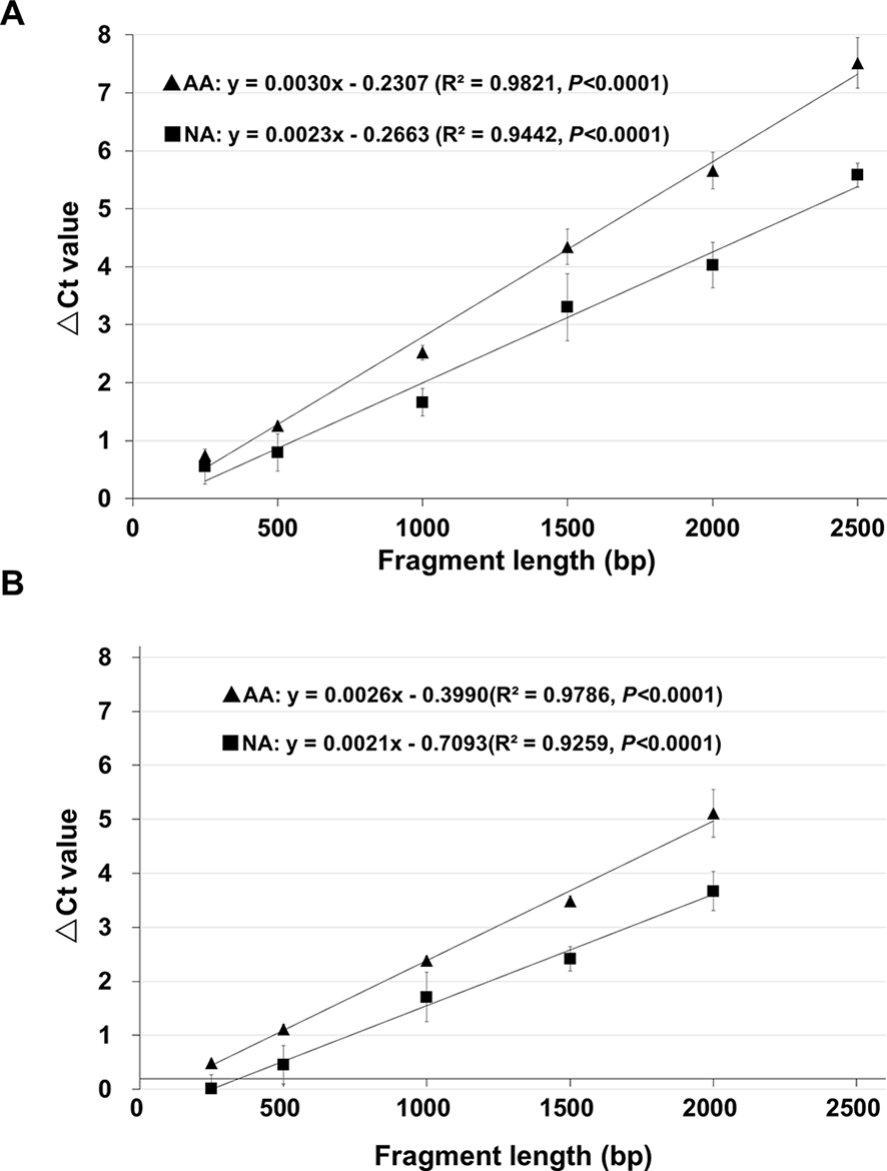
The relationship between ΔCt value and cDNA fragment size analyzed using the same genes. cDNAs of control (100% germination), AA (artificially aged, 0.5% germination) and NA (naturally aged, 0.5% germination) seeds were used to analyze different fragments of the B16 and B20 genes. Each ΔCt value was based on three biological repeats. **(A)** Correlation between the ΔCt value and cDNA fragment length for six cDNA fragments (starting from the STOP codon) of the gene B16. **(B)** Correlation between the ΔCt value and cDNA fragment length for five cDNA fragments (starting from the STOP codon) of the gene B20. Linear regressions were obtained with Microsoft Excel 2010 and all have a P <0.0001. ▲: ΔCt values for AA seeds, ▪: ΔCt values for NA seeds.

Two genes, B16, and B20, (**Table S4**) were used to further determine the relationship between the ΔCt value cDNA fragment length, since they showed a good level of stored mRNA abundance in unaged seeds (**Figure 1**) and both had a coding region more than 2 kb (thus allowing various fragments up to 2,500bp to be analyzed). Using the unaged control, NA (0.5% germination) and AA (0.5% germination) seeds, cDNA regions of 250, 500, 1,000, 1,500, 2,000, and 2,500bp (starting from the STOP codon and counting towards the 5′-direction of an mRNA; **Figure S3**) were analyzed, with three biological repeats for each aging treatment. For both stored mRNAs, there was a very tight correlation between the ΔCt value and cDNA fragment length (with an R^2^ value > 0.90 and P<0.0001) (**Figure 3**), clearly indicating that the chance for a damage or lesion to occur to an mRNA fragment increases with the fragment length.

### Analysis of the Relationship Between the Decrease in Stored mRNAs and Seed Germination

Since seed aging status is traditionally assessed by germination, we determined the relationship between the decrease in stored mRNAs (in ΔCt value) and seed germination. First, NA seeds were used (**Table S1**). Since they were collected at different times, variation in the parental plants and seed quality could affect the speed of seed aging. Their aging status was thus based on seed germination percentage. For the qPCR analysis, we used the 2,000bp fragment of B16 since a longer fragment showed a greater change in ΔCt in aged seeds and thus is more sensitive to seed aging. The ΔCt value increased with decreasing seed germination percentage, but it did not follow a linear pattern (**Figure S4A**). Similar results were obtained with NA seeds (**Figure S4B**).

Determining the relationships among seed aging time, seed germination and the change in stored mRNAs (as reflected by ΔCt value) requires seeds of a similar quality and aged constantly. Accordingly, we used seeds artificially aged under the same conditions. As shown in **Figure S5**, the germination percentage of those seeds decreased non-linearly. Under the aging conditions used, for the first 6 days the change was very slow and for the next 6 days the germination percentage decreased from above 80% to about 10%. After 14 days, the seeds completely lost the ability to germinate. The seed germination curve in **Figure S5** fits well with the generalized model of seed aging, consisting of (1) an asymptomatic phase during which the ability of seeds to germinate changes relatively little, (2) a phase of “rapid mortality” and (3) the last phase in which seeds could no longer germinate (Walters et al., 2010).

### An Improved Quantitative Method for Determining the Relationship Between ΔCt Value of a Stored mRNA and Seed Aging Time

**Figure 4:**
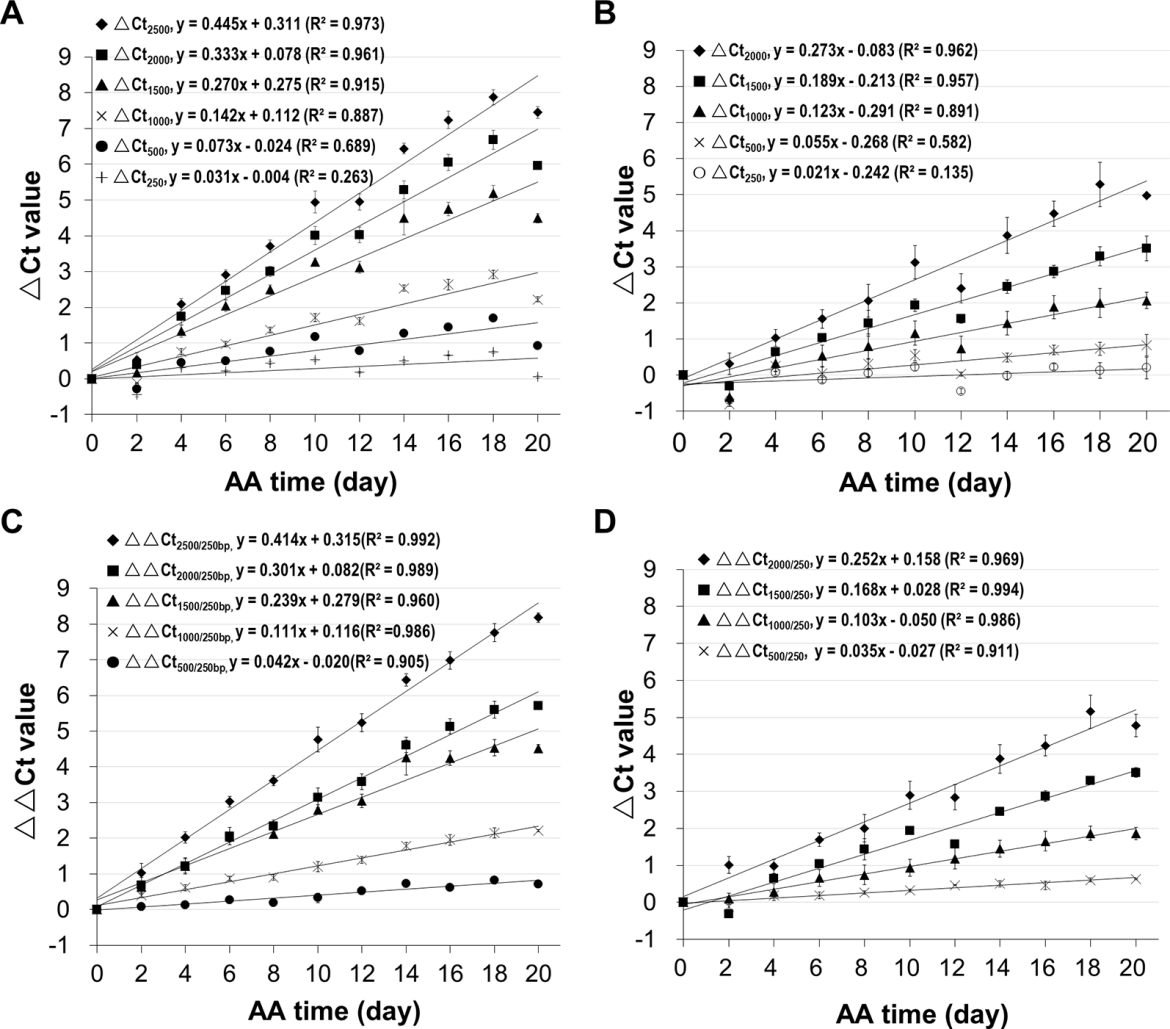
Correlation of mRNA degradation with seed aging time analyzed using different cDNA fragment lengths of two genes. cDNAs of control and artificially aged (AA) seeds for the indicated aging time (days) were used. Different fragments of the gene B16 or B20 were used. ΔCt values are based on three technical repeats. **(A)** Correlation between ΔCt values for six different fragments of B16 and aging time. The subscript after ΔCt indicates the cDNA fragment length (as ΔCt2,500 indicates the ΔCt is for the 2,500bp fragment). **(B)** Correlation between ΔCt values for five different fragments of B20 and aging time. **(C)** Correlation between normalized ΔCt values for six different fragments of B16 and aging time. The normalized ΔCt or ΔΔCt2,000/250bp = ΔCt2,000bp − ΔCt250bp. **(D)** Correlation between normalized ΔCt values of different fragments of B20 and aging time. Linear regression functions were calculated with Excel 2010 and all have a P <0.01 (except for the 250bp fragment of B16 and B20).

Since we know precisely the aging status of the AA seeds (**Figure S5**), we used the AA seeds to evaluate the relationship between ΔCt value and aging time. Also, since the ΔCt value from qPCR analysis depended on the fragment length, we compared different mRNA fragments (starting from the STOP codon position) of B16 and B20. The ΔCt values for different fragments correlated with aging time and interestingly the correlation coefficient increased with the cDNA fragment length (**Figure 4**). The correlation coefficients for fragments longer than 1500bp were close to or greater than 0.90. The slope of the ΔCt regression lines also increased gradually with the increase in fragment length. Thus, the ΔCt values for all long fragments (1500bp or longer) correlated highly with the aging time.

In qPCR analysis, to minimize the differences in template amounts, a gene with a constant expression level is used as an internal control to normalize the Ct value (Livak and Schmittgen, 2001). Since all stored mRNAs analyzed undergo degradation, an mRNA different from the one being analyzed would not serve as a good reference for normalization, and thus a different approach is needed. Because the level of a shorter fragment changes much less than a longer fragment, we reasoned that a relatively short fragment could be used as an internal reference to normalize the Ct value of a larger fragment. In our analysis, a 250bp fragment was used for normalization. This approach is similar to the ΔΔCt method (Livak and Schmittgen, 2001) except that a short fragment from the same mRNA is used as the reference.

Using the B16 2,000bp fragment as an example, the ΔCt value was calculated from the Ct value of the aged sample minus the Ct value of unaged control resulting in ΔCt_2,000(a-c)_. Similarly, the ΔCt value for the short 250bp fragment was calculated from the perspective Ct values for aged and control samples, resulting in ΔCt_250(a–c)_. The normalized ΔCt (or ΔΔCt) was obtained as follows: ΔΔCt_2,000/250_ = ΔCt_2,000(a–c)_ − ΔCt_250(a–c)_. In these ΔCt designations, the fragment lengths are indicated since the ΔCt value depends on the fragment length, and subscripts 2,000/250 indicate the 2,000bp fragment being normalized with the 250bp fragment. The ΔΔCt values were calculated using the data presented in **Figures 4A, B**. As shown in **Figures 4C, D**, the correlations improved for all fragments, showing that normalizing the ΔCt values using a much shorter fragment reduced data variation.

**Figure 5:**
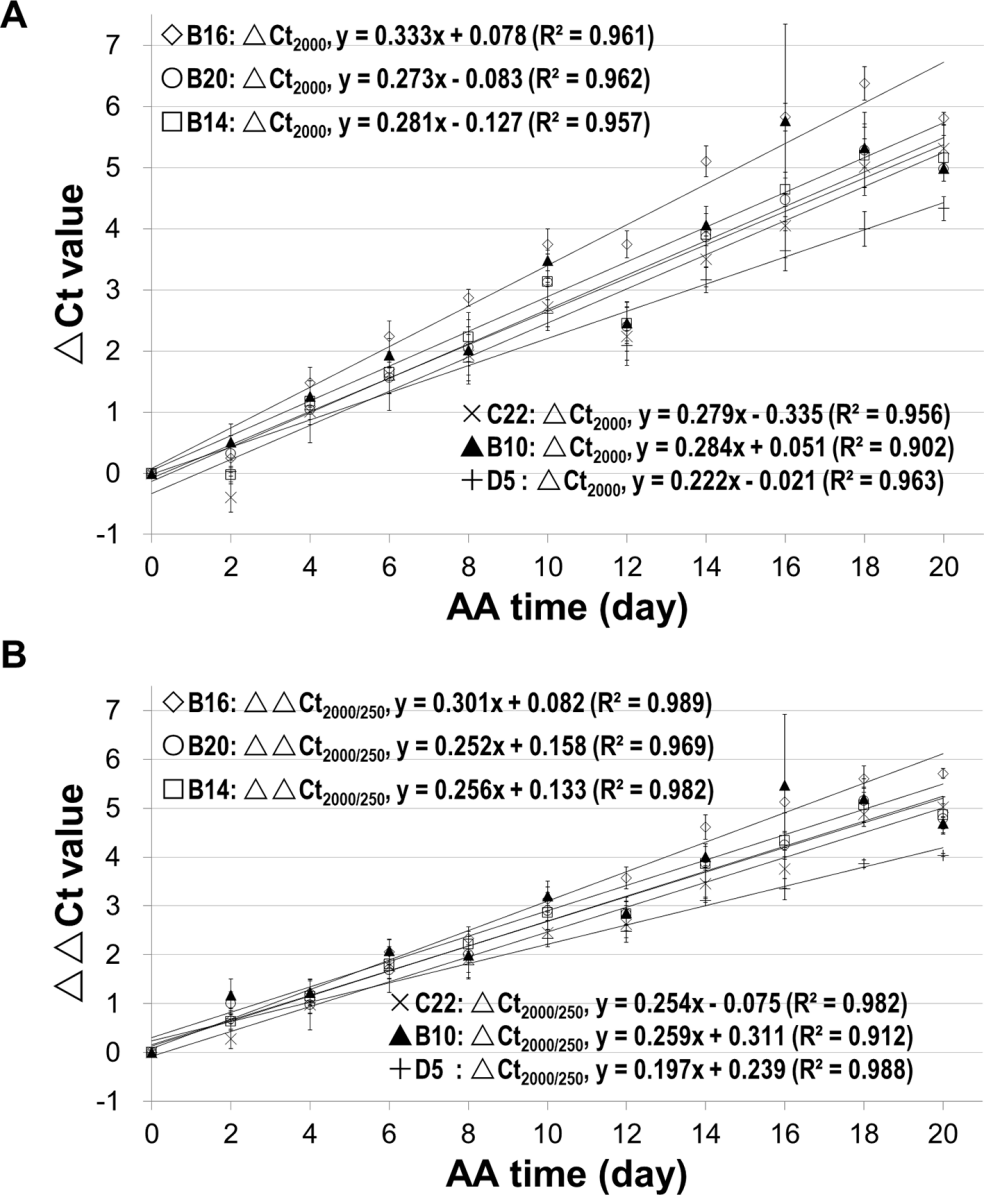
Relationship between the ΔCt values from analyzing stored mRNAs of six genes and seed aging time. cDNAs of control and artificially aged (AA) seeds for the indicated time (days) were used. For each of the six genes (B16, B20, B14, C22, B10 and D5), a 2,000bp cDNA fragment was analyzed. ΔCt = Ct of the aged sample – Ct of control. All ΔCt values are the averages of three biological repeats. **(A)** Relationship between ΔCt value of the 2,000bp fragment (ΔCt2,000) and AA aging time for the six genes. **(B)** Relationship between normalized ΔCt value of the 2,000bp fragment (ΔΔCt2,000/250) and AA aging time. ΔΔCt2,000/250bp = ΔCt2,000bp − ΔCt250bp. Linear regressions were obtained with Microsoft Excel 2010 and all have a P < 0.0001.

To assess changes in different stored mRNAs, stored mRNAs of six genes were analyzed using a 2,000bp fragment. As shown in **Figure 5**, the unnormalized ΔCt value correlated highly with aging time for all six genes. Still, after the normalization, the correlation coefficients were further improved (**Figure 5B**). These results show that the normalized ΔCt values have an improved correlation with seed aging, and any of the six stored mRNAs could serve as an informative biomarker to assess seed aging.

### Estimating the Relative Amount of Undamaged Stored mRNAs During Seed Aging

Although the ΔCt (or ΔΔCt) values highly correlate with seed aging time, they do not show directly the changes in the level of an mRNA during seed aging. We derived the following equation to estimate the relative amount of undamaged stored mRNAs based on the qPCR data (see equation derivation in *Methods*):

(3) $${\text{N}}_{\text{a}}/{\text{N}}_{\text{c}}=1/2^{\Delta \text{Ct}(\text{a}-\text{c})},$$

where

N_a_ = cDNA copy number of the aged sample, and

N_c_ = cDNA copy number of the unaged control.

For instance, for B16_2,000/250_, the percentage of undamaged mRNA in aged seeds could be estimated by substituting “ΔCt(a–c)” in the above equation with the linear regression function for B16_2,000/250_, as follows:

$${\text{N}}_{\text{a}}/{\text{N}}_{\text{c}}*100\%=1/2^{0.301x+0.082}*100\%$$

where *x* refers to AA time in days.

**Figure 6:**
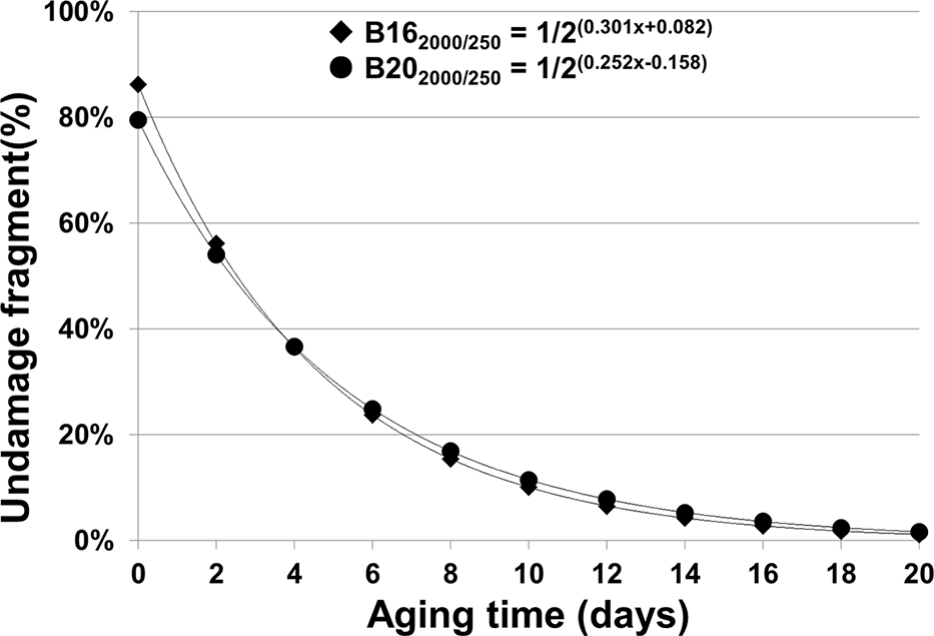
Percentage of estimated undamaged mRNAs of B16 and B20 in seeds with different artificial aging time (days). We have derived mathematical equations to estimate the relative amount or percentage of undamaged stored mRNAs based on the ΔCt regression functions. The regression functions for different fragments of B16 and B20 are from **Figures 3C, D**. For B162,000/250bp, the percentages at different aging times were calculated based on the equation: y = 1/2ΔCt(B162,000/250bp) × 100% = 1/2(0.301+ 0.082)100%, while for B202,000/250bp, the equation is y = 1/2ΔCt(B202,000/250bp) × 100% = 1/2(0.252+ 0.158) 100%.

Thus, the percentages of undamaged B16_2000/250_ and B20_2000/250_ templates in aged seed samples were estimated based on the ΔCt values from **Figure 5B** and showed an exponential decrease over the aging time (**Figure 6**).

### Estimating the Rate of Stored mRNA Degradation at One Nucleotide Level

To further understand the characteristics of stored mRNA degradation, we established a method for estimating the rate of mRNA degradation at one nucleotide level. As described in Methods, the probability of an mRNA of “*n*” nucleotides with “0” break after “*t*” days of AA treatment can be estimated as:

(7) $$P(0)=e^{-t\beta n}\approx {\text{N}}_{\text{a}}/{\text{N}}_{\text{c}}=1/2^{\Delta \text{Ct}},$$

where *β* is the frequency of break per nucleotide per day.

Taking a logarithmic transformation of equation (7), we have:

(8) $$\beta =\text{ln}(2^{\Delta \text{Ct}})/tn$$

We can use B16_2000/250_ fragment of the 18-day AA treatment as an example. Since the ΔCt value for B16_2000/250_ was normalized with the ΔCt value of B16_250_, it reflects the changes for the fragment length of 2,000bp–250bp = 1,750bp, and thus the number of nucleotides *n* = 1,750. The aging time *t* = 18 days, and the normalized ΔCt value was 5.60. Based on equation (8), we have:

$$\beta =\ln(2^{5.60})/18\times 1750=1.23\times 10^{-4}$$

Thus, the probability for one lesion to occur is 1.23 × 10^−4^ per nucleotide per day for the B16 fragment under the current AA conditions. Similarly, we obtained the *β* values for other fragments of B16 and B20 and aging times (**Table 1**). Remarkably, the *β* values were similar at different aging times for different fragment sizes of B16 or B20, indicating that the two stored mRNAs degraded at a constant rate over the aging time analyzed. Regarding different stored mRNAs, the mRNAs of B16 and B20 also degraded at similar rates, as shown by the total averages of the β values (1.22 × 10^−4^ vs 0.94 × 10^−4^) (**Table 1**). Further, the *β* values for the stored mRNAs of six genes (**Figure 5B**) confirm, after an ANOVA test, that they generally degraded constantly over aging time, and with similar rates to each other (**Table 2**).

**Table 1:** Estimated “*β*” values for different fragments at different aging times.

Gene code	Fragment^(1)^	Fragment size (bp)	*β* value (break per nucleotide per day x 10^-4^)^(2)^						
			Day 4	Day 8	Day 12	Day 16	Day 20	Average	Overall
B16	B16_2500/250bp_	2,250	1.56+0.12	1.39+0.06	1.34+0.06	1.34+0.05	1.26+0.02	1.38+0.04	1.22±0.03
	B16_2000/250bp_	1,750	1.21+0.22	1.16+0.09	1.18+0.07	1.27+0.05	1.13+0.02	1.19+0.04	
	B16_1500/250bp_	1,250	1.69+0.27	1.46+0.08	1.41+0.09	1.47+0.07	1.25+0.03	1.46+0.06	
	B16_1000/250bp_	750	1.42+0.21	1.04+0.09	1.07+0.08	1.13+0.09	1.02+0.02	1.13+0.06	
	B16_500/250bp_	250	0.88+0.20	0.65+0.30	1.22+0.06	1.06+0.07	0.98+0.01	0.96+0.08	
	Average		1.35+0.11	1.14+0.10	1.24+0.04	1.25+0.05	1.13+0.03		
B20	B20_2000/250bp_	1,750	0.97+0.19	0.99+0.18	0.94+0.11	1.05+0.07	0.95+0.06	0.98+0.05	0.94±0.04
	B20_1500/250bp_	1,250	0.81+0.15	0.96+0.20	0.93+0.03	0.91+0.05	0.92+0.04	0.91+0.05	
	B20_1000/250bp_	750	0.64+0.52	0.86+0.31	0.91+0.22	0.96+0.15	0.86+0.08	0.85+0.12	
	B20_500/250bp_	250	1.36+0.38	0.94+0.16	1.09+0.01	0.80+0.20	0.88+0.02	1.01+0.09	
	Average		0.95+0.17	0.94+0.09	0.97+0.06	0.93+0.06	0.90+0.02		

We have derived an equation to estimate the probability of producing a break in the cDNA of a stored mRNA at one nucleotide level, which we refer to as the “β” value (see Methods). It can be estimated based on the ΔCt values of qPCR analysis. Each “β” value is based on three technical repeats, and the standard error is indicated.

^(1)^The fragment analyzed and fragment used for normalizing the ΔCt value are indicated. For instance, B16_2500/250bp_ indicates that a 2,500bp fragment of B16 was analyzed and normalized with the ΔCt of a 250bp fragment.

^(2)^“β” value was estimated based on the ΔCt of the AA seed sample at the indicated aging time, and is the probability of having a break per nucleotide per day.

**Table 2:** Estimated “*β*” values for stored mRNAs of six genes at different aging times.

Fragment^(1)^	*β* value (break per nucleotide per day × 10^−4^)					
	Day 4	Day 8	Day 12	Day 16	Day 20	Average
B16_2000/250bp_	1.21±0.22	1.16±0.09	1.18±0.07	1.27±0.05	1.13±0.02	1.19±0.04
B20_2000/250bp_	0.97±0.19	0.99±0.18	0.94±0.11	1.05±0.07	0.95±0.06	0.98±0.05
B10_2000/250bp_	1.21±0.26	0.99±0.10	0.94±0.08	1.35±0.36	0.93±0.02	1.09±0.09
B14_2000/250bp_	1.14±0.11	1.09±0.18	0.94±0.08	1.08±0.04	0.96±0.07	1.04±0.04
C22_2000/250bp_	0.97±0.51	0.95±0.20	0.87±0.12	0.93±0.10	1.00±0.04	0.94±0.10
D5_2000/250bp_	1.00±0.20	0.89±0.13	0.82±0.04	0.83±0.05	0.80±0.01	0.87±0.05

The β value refers to the probability of producing a break in the cDNA of a stored mRNA at one nucleotide level. The β values for stored mRNAs of six genes at different aging times were estimated based on the ΔCt values of qPCR analysis, as shown in **Figure 5B**. Each “β” value is based on three biological repeats, and the standard error is indicated.

^(1)^The fragment analyzed and fragment used for normalizing the ΔCt value are indicated. For instance, B16_2000/250bp_ indicates that a 2,000bp fragment of B16 was analyzed and normalized with the ΔCt of a 250bp fragment.

### Comparisons with Traditional Methods of Assessing Seed Aging

**Figure 7:**
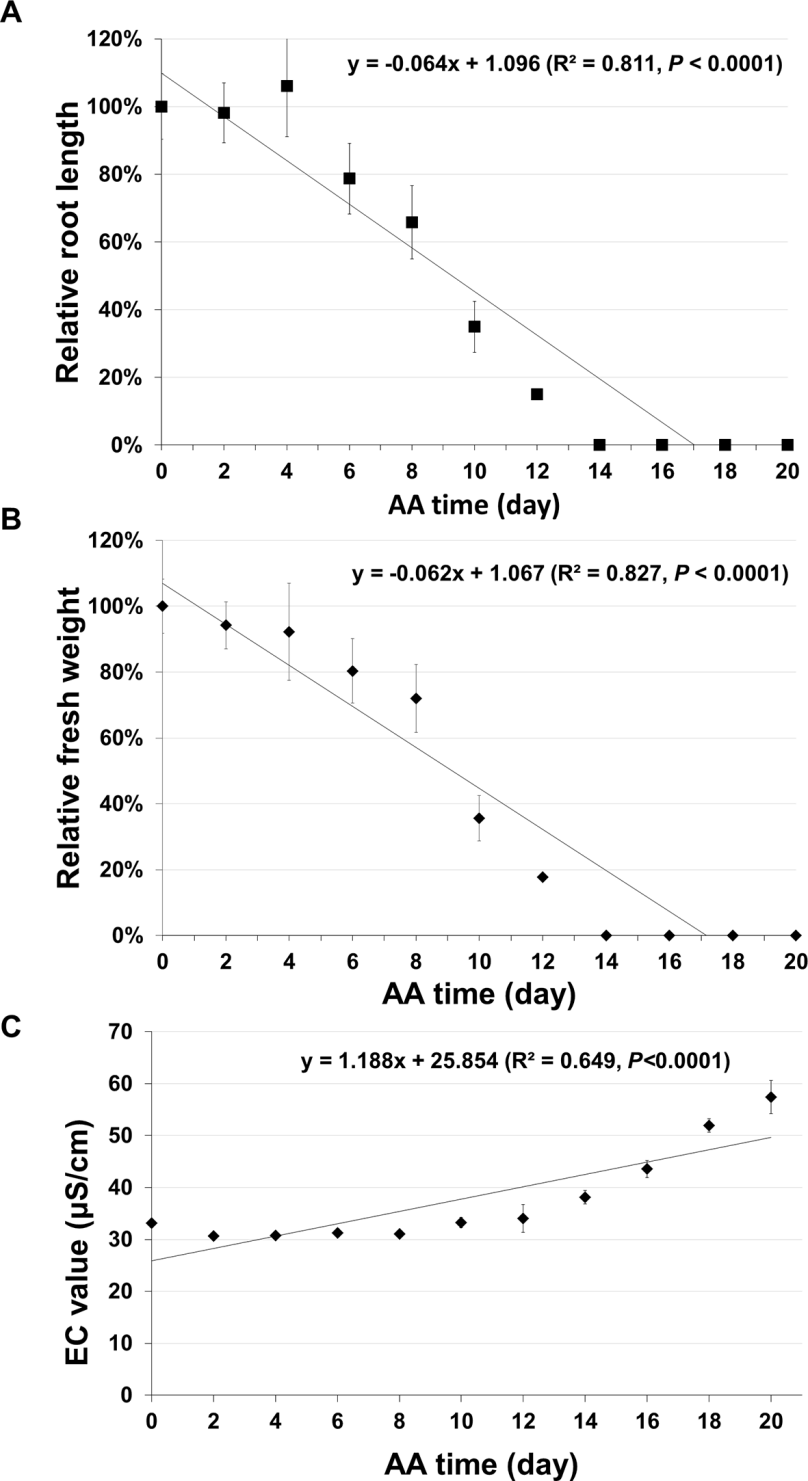
Analysis of seed aging by traditional methods. Artificially aged (AA) seeds were analyzed based on seedling growth and electrical conductivity (EC) test. Two parameters of seedling growth were determined. For each time point, five plates were used and each plate had unaged and aged seeds planted side-by-side. After 10 days of incubation, the relative primary root length and fresh seedling weight (the ratio between aged and control samples) were obtained for each plate. Each datum is based on five plates with the standard error shown. **(A)** Relative primary root length. **(B)** Relative seedling fresh weight. **(C)** EC value. Seeds (10 mg) of different aging times (with three biological repeats for each time point) were soaked in 1.5 ml ddH2O used and EC analysis was performed after 16 hours of incubation. Linear regressions were obtained with Microsoft Excel 2010 and had a P < 0.0001.

In addition to seed germination, other traditional methods have also been used for assessing seed aging. Two common ones are seedling growth (Marcos Filho, 2015) and electrical conductivity (EC) (Matthews and Powell, 2006; Ramos et al., 2012). The EC test is based on the principle that aged seeds leak more electrolytes in water than unaged seeds. For seedling growth, we analyzed the root growth (length) and fresh seedling weight. For each aging time point five plates were used, and in each plate, seven unaged and seven aged seeds were planted side by side. After 10 days of incubation, the root length and seedling fresh weight were determined. The root length (**Figure 7A**) and seedling fresh weight (**Figure 7B**) decreased gradually to zero with seed aging time. In both cases, the curves followed a similar pattern to that of seed germination. They could not precisely distinguish seeds aged for two to six days and failed to detect any further changes in seeds aged for more than 14 days. For EC test, the data had a correlation with seed aging time, but with a much lower coefficient than that of the ΔΔCt value for the stored mRNAs (**Figure 7C**). Therefore, the qPCR method based on the degradation of a stored mRNA is a more accurate method to assess seed aging than the three classical methods.

## Discussion

### Degradation of Stored mRNAs is Highly Correlated with Aging in Naturally and Artificially Aged Arabidopsis Seeds

It has been reported that the integrity of rRNAs decreases in aged seeds of several plant species based on the appearance of smaller RNA bands on the electropherograms (Brocklehurst and Fraser, 1980; Thompson et al., 1987; Reuzeau and Cavalie, 1997; Matthews and Powell, 2006; Kranner et al., 2011; Sharma et al., 2018). However, it was difficult to quantify the relationship between RNA band changes and seed aging time. Recently, Fleming et al. (2017) examined the changes in RNA integrity using the RNA integrity number (RIN). The RIN is an index based on multiple features of electropherograms generated from Agilent Bioanalyzer, including the total RNA ratio, the heights of the 25S and 18S peaks, and the “fast area” (i.e. small RNA molecules/fragments) ratio (to the total area of the electropherogram) (Imbeaud et al., 2005; Schroeder et al., 2006). Using soybean seeds stored up to 27 years, a high correlation between the RIN of RNA isolated from cotyledons and the percentage of germination was observed, but the correlation was lower between the RIN of the RNAs from the embryonic axis and percentage of germination (Fleming et al., 2017). The authors also extended this analysis to several other plant species and observed similarly that the RNA integrity decreased during seed aging (Fleming et al., 2018a).

We also analyzed our Arabidopsis RNA samples using Agilent Bioanalyzer. At the start of this study, we had optimized the RNA isolation protocol to ensure the quality and consistency of RNA samples. The RINs were very similar (mostly between 8–9) among samples of different aging times (**Table S5**). Thus, for the Arabidopsis RNA samples in this study, the RIN value was not nearly sensitive and effective for assessing seed aging compared to the ΔCt value of a stored mRNA. Since the RIN is an integrated parameter based on multiple features of a RNA electropherogram, it is affected not just by RNA degradation, but also the purity, concentration and abnormal peaks. Furthermore, the method developed here is well suited to analyze the changes of individual RNAs, which the RIN data could not determine.

Much less research has been done regarding stored mRNAs and seed aging. The transcripts of an Arabidopsis gene, *At3g08030*, and its homologs in two other plant species were found to be present in freshly collected seeds, but reduced or not detected in aged seeds (Garza-Caligaris et al., 2012). The authors suggested that the *At3g08030* transcript could be a potential biomarker for seed aging. However, the quantitative relationship of the *At3g08030* transcript level with aging time or seed germination frequency was not determined. Recently, Fleming et al. (Fleming et al., 2018b) observed fragmentation of stored mRNAs in soybean embryonic axis by transcriptomic analysis and suggested that mRNA breaks occur at random positions.

The intactness of an mRNA is reflected by its ability to be reverse-transcribed into a cDNA. A break or damage to a nucleotide within the mRNA region which blocks cDNA synthesis will result in a partial cDNA. The cDNAs were synthesized from the polyA tail using an oligo dT primer and we typically use the STOP codon as the start point (**Figure S3**). Since the relative amount of mRNA can be determined by qPCR using cDNA as the template, the difference between the Ct of an aged sample and Ct of the unaged control would reflect the change in the amount of the undamaged mRNA for the aged sample relative to the control.

Our RT-PCR analysis showed that almost all stored mRNAs initially analyzed (over 80 of them) showed a gradual decrease with longer stored mRNAs decreasing faster. qPCR analysis of 29 mRNAs showed that the extent of decrease closely correlated with the mRNA coding region length. When six stored mRNAs were analyzed, it was revealed that the decreases of their levels were highly correlated with the aging time and at similar rates. These results collectively demonstrate that the degradation of Arabidopsis seed stored mRNAs as represented by the ΔCt value is highly correlated with the aging time. In addition, the observation that the degradation of stored mRNAs was very similar in AA and NA seeds with the same germination percentage (0.5%) suggests that the AA seeds aged in a similar way to the NA seeds even although the degradation of stored mRNAs in NA seeds took a much longer time (years) to occur.

The proteomic analysis suggested that the loss in seed germination ability could be partly attributed to the inability of the aged seeds to produce a normal proteome during germination (Rajjou et al., 2008). Thus, it is possible that degradation of seed stored mRNAs would reduce the amount of template for synthesizing proteins that are critical for successful germination (Rajjou et al., 2008). As analyzed in this study, genes in Group A and B encode proteins involved in ubiquitnation and HSPs. Protein ubiquitination and HSPs play fundamental roles in many processes (Callis, 2014; Fragkostefanakis et al., 2015; Shu and Yang, 2017). It has been reported that HSPs could enhance seed longevity in several plant species such as rice (OsHSP18.2, Kaur et al., 2015) and tobacco (HaHSFA9, (Prieto-Dapena et al., 2006). We showed that the stored mRNAs of almost all the ubiquitination-related and HSP genes analyzed decreased gradually in the aged seeds (**Figure 1B**). The proteomic analysis also suggested that, for germinating rice seeds treated by mRNA synthesis inhibitor Act D, proteins and enzymes involved in energy production and cell structure maintenance could be produced with stored mRNA providing templates (Sano et al., 2012). In this study, D17 and D19 encode enzymes participating in pathways related to energy production such as malate metabolism and the oxidative pentose phosphate pathway, while C26 and C27 encode two tubulin proteins which are structural components of cytoskeleton. The degradation of these stored mRNAs in aged seeds could affect the amount of stored mRNA templates for synthesizing the proteins and deter seed germination. Although the general effect of stored mRNA degradation on the initial protein synthesis in seed germination can be easily understood, it needs to be pointed out that it is still an open question regarding which stored mRNAs are required for seed germination. Addressing this question would require specific elimination of the stored mRNAs of a particular gene without affecting other stored mRNAs and processes, which is difficult to achieve technically.

### A New Approach of Normalizing qPCR Data Improves the Analysis of Stored mRNAs

In qPCR analysis, a reference gene is typically used to normalize the ΔCt value (Livak and Schmittgen, 2001). Since all stored mRNAs showed degradation in seed aging, we used a short fragment (e.g. 250bp) for normalization. The results show that this approach was effective and improved the correlations between the ΔCt value and aging time for different fragment lengths (**Figure 4**) and for different genes (**Figure 5**).

This method should be applicable for studying seed stored mRNAs in different plants. The long and short fragments do not have to be the same in lengths as the ones used here. For a different application, it would be good to determine the quantitative relationship between the fragment length and aging time (**Figure 4**), based on which suitable long and short fragments could be selected.

### Quantitative Methods are Developed to Estimate the Relative Amount of Intact mRNAs and Rate of mRNA Degradation at One Nucleotide Level

We observed two fundamental characteristics of stored mRNA degradation during seed aging. First, when different fragment lengths of a stored mRNA were compared at a given aging time point the ΔCt value increased linearly with the mRNA length (**Figure 3**). This finding indicates that when the mRNA length increases by the same increment (e.g. 500bp), its level decreases by a similar extent (indicated by a similar increment in ΔCt value), suggesting that the damage or degradation of a stored mRNA occurs randomly along the length of a stored mRNA. Second, when a given length (e.g. 2,000bp) of a stored mRNA was considered, the ΔCt value highly was correlated with seed aging time (**Figure 4**), indicating that the time for the mRNA level to decrease by 50% (ΔCt = 1, assuming 100% PCR amplification efficiency) is constant or in another word a stored mRNA degrades at a constant rate over the aging time. Based on these two characteristics, we can estimate the relative amount of undamaged mRNAs at a given aging time and frequency of degradation at one nucleotide level.

The relative amount of undamaged mRNA level could be estimated by substituting ΔCt(a–c) in the equation N_a_/N_c_ = 1/2^ΔCt(a–c)^ with the linear regression function for the stored mRNA or a given fragment (see Methods). The percentages of estimated undamaged mRNAs, e.g. for the B16 and B20 2,000bp fragments as shown in **Figure 6**, provide us with a clearer understanding regarding how stored mRNA levels change over aging time.

We further developed a method to estimate the average rate of degradation at one nucleotide level. A similar approach has been used to estimate the average lesion per DNA strand in DNA damage analysis (Yakes and Vanhouten, 1997; Ayala-Torres et al., 2000). We derived a formula (*β* = ln(2^ΔCt^)/*tn*) to estimate the average frequency of “breaks” per nucleotide per day, which we named as “*β* value”. Interestingly, the *β* values remained fairly constant throughout the aging time for different fragments of the same stored mRNA (**Table 1**) and for different stored mRNAs (**Table 2**), indicating that a stored mRNA is degraded at a fairly constant rate under the present aging conditions. The rates of degradation for six different mRNAs were also very similar.

To our knowledge, there has been no previous report on quantifying the degradation of stored mRNAs during seed aging. Understandably, the *β* value may depend on specific aging conditions. The *β* value thus provides a new, simple and quantitative parameter for analyzing stored mRNA degradation and seed aging. It should allow comparisons of different mRNAs, different regions of the same mRNA, and different aging conditions in plant seeds.

Our quantitative analyses of individual stored mRNAs showed that each stored mRNAs was degraded at a constant rate over the aging time, while among different stored mRNAs analyzed they showed similar rates of degradation. Since all stored mRNAs analyzed in this study showed similar trends of gradual decreases, these results suggest a scenario that majority of stored mRNAs are degraded with a similar pattern and at a constant rate during seed aging. Given that there are at least 12,000 stored mRNAs in Arabidopsis seeds (Nakabayashi et al., 2005), an interesting question is whether differences exist among stored mRNAs in terms of the rate of degradation. The scale of work prevented us from comparing the rate of degradation for a large number of individual stored mRNAs in this study. The methods developed in this study should help to address this question through comparative and quantitative analyses of individual stored mRNAs, which had been difficult to determine previously.

### Stored mRNAs can Serve as more Precise Biomarkers for Monitoring Seed Aging

In assessing seed aging status, the classical methods such as seed germination percentage and seedling growth have a few major problems: little change during the asymptomatic phase and the lack of strictly linear relationship between in the parameter analyzed and aging time. A better parameter should have a tight linear relationship with seed aging time and be able to detect changes during the asymptomatic phase (Fu et al., 2015). Our results show that the ΔCt for one stored mRNA is highly correlated with seed aging time. Thus, the changes in stored mRNAs (represented by ΔCt values) can serve as a more precise method of assessing seed aging. In addition to the qPCR-based methods, long-read DNA sequencing technologies (Lu et al., 2016; Oikonomopoulos et al., 2016) may also be used for determining the changes in transcript length. However, analyzing one or a few mRNAs is technically much simpler and requires less effort, cost and time than sequencing entire cDNA libraries. Further, technically, multiple steps of DNA manipulation including rounds of DNA purification are needed for cDNA library preparation, for instance for MinION sequencing (https://community.nanoporetech.com/protocols). The fragment size distribution in the resulting cDNA libraries may not truly represent the fragment size distribution in the initial RNA samples due to the differences in the binding efficiency of different DNA fragment sizes to the DNA purifying matrix, affecting the quantitative analysis based on the cDNA fragment lengths.

Since seeds age with different speeds under different conditions, seed storage time may not be a good indicator of the seed aging status. Stored mRNAs can serve as more reliable biomarkers. We observed that the AA and NA seeds with 0.5% germination frequency showed similar characteristics in stored mRNA integrity (**Figure 3**) despite the huge differences in their aging time. Thus, one possible way of measuring the aging status of different NA seeds is to establish a reference aging timeline using AA seeds, and then map the NA seed samples to a point on the reference timeline, in a way similar to the use of a reference protein to determine the concentrations of protein samples. Thus, stored mRNAs could provide a more reliable and precise yardstick for determining seed aging status and facilitate seed aging research. On the other hand, this method requires molecular biology expertise and equipment, which is technically more challenging and costs more compared to the traditional seed germination assay. The traditional methods such as seed germination assay could still provide reasonable indication of seed aging. Thus, these methods may still be preferred for seed quality analysis laboratories, as they cost much less and require no molecular biology expertise and equipment. However, with time, molecular techniques might become simpler and more streamlined making the analysis of stored mRNA degradation easier to perform.

In this study, we developed new methods to quantify the changes in seed stored mRNAs and estimate the rate of mRNA breakdown at one nucleotide level. These methods should facilitate the studies of seed stored mRNAs in plants. The *β* value or frequency of breaks per nucleotide per day should make it easier to quantify the effects of different conditions on stored mRNAs and seed aging. Furthermore, these methods should also be applicable for analyzing slow RNA degradation in other plants and non-plant systems.

## Experimental Procedures

### Plant Growth

*Arabidopsis thaliana* (ecotype “Columbia”) plants were grown in pots placed in a growth room (20°C constant, 16/8 h day/night cycles with a daylight fluorescent white light of 100 ± 15 μmol m^−2^ s^−1^). Harvested seeds were air-dried at room conditions and then stored in 2 ml microtubes with airproof screw caps under 4°C.

### Seed Aging Treatments

For natural aging, Arabidopsis seeds collected at different times (**Table S1**) were stored at 4°C. For accelerated aging treatments, a procedure described previously (Sugliani et al., 2009; Marcos Filho, 2015) was used with modifications. Dry seeds were placed in 2 ml tubes with the cap removed (each tube having 100 mg seeds). The tubes were placed into a plastic container with saturated KCl solution at the bottom (resulting in air humidity about 83% at 37°C). The container was closed with a lid, sealed with Parafilm, and placed at 37°C. Following the treatment, the seed samples were removed, air-dried for 3 days at room temperature and stored at 4°C in microtubes with the tube caps closed.

### Analyses of Seed Germination, Seedling Weight and Root Length

Seeds were surface-sterilized and stored for 2 days at 4°C in the dark for stratification. They were sown on medium containing ½-strength of Murashige and Skoog salts (Murashige and Skoog, 1962), 1% w/v sucrose and 0.7% w/v agar. The plates were placed in a growth chamber (16h/8h photoperiod and light intensity of 90 ± 10 *μ*mol m^−2^ s^−1^) at 20°C. After 7 days, a seed was considered germinated if the radicle was equal or longer than the length of the seed. For the fresh seedling weight, all seedlings from one plate were weighed together and the average seedling weight was calculated. For the root length, seeds were sown on the plates, which were placed vertically in the growth chamber. After 10 days, plate images were taken, the primary root length of each seedling was measured with the NIH ImageJ software (Version 1.42) and the average root length per plate was calculated. At least three plates were used for each seed sample.

### Isolation of Total RNA

Total RNA was isolated from dry seeds using a protocol (Onate-Sanchez and Vicente-Carbajosa, 2008) with some modifications. In brief, seeds were grounded in liquid nitrogen. The powder was added to a mixture of 550 µl extraction buffer (0.4 M LiCl, 0.2 M Tris (pH 8.0), 25 mM EDTA, 1% SDS) and 550 µl chloroform in a 1.5 ml tube. The content was well mixed with handshaking. Following a 3 min centrifugation, the upper phase (500 µl) was transferred to a new 1.5 ml tube containing 500 µl phenol. After a simple vortex and 5 min room temperature incubation, 200 µl chloroform was added. Following a 3-min centrifugation, the upper phase (500 µl) was transferred into a new 1.5 ml tube containing 170 µl 8 M LiCL. After 30-min storage in a −20°C freezer, the mixture was centrifuged for 15 min at 4°C. The supernatant was discarded and the pellet was further treated with DNase. The DNase treated mixture was transferred into a solution containing 500 µl DEPC-H_2_O, 250 µl 100% ethanol and 7 µl 3 M NaAC and centrifuged for 10 min at 4°C. The supernatant was further transferred into a solution containing 750 µl 100% ethanol and 43 µl 3M NaAC, and stored at −20°C for 30 min, followed by centrifugation, pellet washing and air-drying. At last, 20 µl DPEC H_2_O was added to resolve the pellet. In this protocol, all centrifugations were performed at 22, 000 g. RNA quantity and purity were determined using a NanoDrop 8000 Spectrophotometer (Thermo Fisher Scientific, www.thermofisher.com). RNA integrity was evaluated with agarose gel electrophoresis and Agilent 2,100 Bioanalyzer (Agilent Technologies, http://www.agilent.com).

### RT-PCR and qPCR Analyses

The first-strand cDNA was synthesized from total RNA using the ThermoScript RT-PCR system according to manufacturer’s instructions (Invitrogen). The cDNA mixture was diluted 1:3 with sterile H_2_O and used in PCR amplification. Genes used for surveying the presence of stored mRNAs by RT-PCR are listed in **Table S2**. PCR products separated by electrophoresis in 1% agarose gels and stained with ethidium bromide. The gel images were obtained with a BioDoc-It imaging document system and used without any modifications (except for cropping to show the DNA band).

qPCR was performed with the Green-2-Go qPCR Mastermix (BioBasic). The mastermix (12.5 µl for each), primers (in the final concentration of 0.25 µM each) and cDNA (1.5 µl for each) were added in the reaction volume of 25 µl each. Reactions were run in the Bio-Rad CFX96 Real-Time system and the threshold cycle (Ct) value for each reaction was generated by CFX Maestro Software (Version 3.0). Linear regression analysis was performed with Excel 2010 and ANOVA was conducted with RStudio (https://www.rstudio.com/).

### Estimating the Relative Amount of Undamaged Stored mRNAs during Seed Aging

Assuming that the qPCR amplification efficiency is nearly 100% for the early phase up to the threshold cycle of qPCR (Ct), the amplified DNA copy number (designated as C here) can be estimated as:

$$\text{C}=\text{N}\cdot 2^{nc}$$

where N is the initial copy number of cDNA (cDNA is the proxy for undamaged mRNA or a selected region of mRNA), and *nc* is the number of PCR cycles used.

For the unaged control sample, the copy number of amplified DNA (C_c_) for a specific fragment of mRNA at the threshold cycle can be estimated as:

(1) $${\text{C}}_{\text{c}}={\text{N}}_{\text{c}}\;\cdot 2^{\text{Ct}(\text{c})},$$

Similarly for the aged sample, the copy number of amplified DNA (C_a_) at the threshold cycle can be estimate as:

(2) $${\text{C}}_{\text{a}}={\text{N}}_{\text{a}}\cdot 2^{\text{Ct}(\text{a})}$$

Since the copy number of DNA at the threshold cycle can be considered the same for the control and aged samples (C_a_ = C_c_), the relative amount of undamaged stored mRNA fragment in aged sample over unaged sample (i.e. N_a_/N_c_) can be estimated as:

(3) $$\begin{array}{l} {\text{N}}_{\text{a}}/{\text{N}}_{\text{c }}=2^{\text{Ct}(\text{c})}/2^{\text{Ct}(\text{a})}\\ =2^{\left[\text{Ct}(\text{c})-\text{Ct}(\text{a})\right]}\\ =1/2^{\Delta \text{Ct}(\text{a}-\text{c})} \end{array}$$

As the variance of this estimator is proportional to the variance of ΔCt(a–c), the standard error of the estimate can be obtained from repeated measurements of the differences in their threshold cycles.

### Estimating the Probability of Stored mRNA Degradation at One Nucleotide Level

Our results suggested that the lesions to stored mRNAs occurred likely randomly. We derived an estimator for the probability of stored mRNA degradation at one nucleotide level. For simplicity, we assume that any change in mRNA to be equivalent to “a break on a nucleotide” if it prevents the mRNA template from being reverse-transcribed at the particular nucleotide. Also, there are only two possible outcomes for an intact nucleotide after a given time of ageing (say days): either it is broken at the probability *β*, or it is still intact at the possibility of 1- *β*. Under these conditions, the probability for a stored mRNA with “*n*” number of nucleotides to have “*x*” number of break(s) would follow a binomial distribution and can be defined as:

(4) $$P(x)=C_{x}^{n}{\left(t\beta \right)}^{x}{\left(1-t\beta \right)}^{n-x}$$

where *P*(*x*) is the possibility that x break(s) occur on the mRNA fragment

*n* is the number of nucleotides in the fragment,

*t* is the ageing time in days, and

*β* is the possibility that a break occurs on each nucleotide per day.

In reverse-transcription using oligo dT as the primer, only the mRNA templates without a break from the 3’ end to the 5’ target position could be reverse-transcribed into cDNAs and be further amplified in qPCR reaction. *P*(0) can be defined as the probability that no nucleotide is broken within the given mRNA template, and thus we have:

(5) $$\begin{array}{l} P(0)={\text{C}}_{0}^{\text{n}}(t\beta {)}^{0}(1-t\beta {)}^{n-0}\\ \;=1*1*(1-t\beta {)}^{n}\\ \;=(1-t\beta {)}^{n} \end{array}$$

For a long fragment, it consists of a large number of nucleotides (with *n* → infinite), and the equation (5) can be simplified following a binomial series expansion as:

(6) $$P(0)=e^{-t\beta n}$$

Since the relative amount of undamaged stored mRNAs (with “0” break) can be estimated from equation (3), we can have the following to estimate *P*(0):

(7) $$P(0)=e^{-t\beta n}\approx {\text{N}}_{\text{a}}/{\text{N}}_{\text{c}}=1/2^{\Delta \text{Ct}}$$

Taking a logarithmic transformation of equation (7), we can estimate the probability (*β*) for one nucleotide to break in an mRNA of *n*-nucleotide long and aged for *t* time, as:

(8) $$\beta =\ln(2^{\Delta Ct})/tn$$

### Electrical Conductivity (EC) Test

Ten mg seeds were soaked in 1.5 ml ddH2O in a 15 ml tube. After 16 h incubation at room temperature (22°C), the EC was measured with VWR Bench/Portable Conductivity Meter (VWR, www.vwr.com) using three repeat seed samples.

## Data Availability Statement

All datasets generated for this study are included in the article/**Supplementary Material**.

## Author Contributions

HW and Y-BF initiated the project. LZ performed most of the experimental work. SW contributed to the initial RNA isolation and cDNA synthesis. LZ, HW, and Y-BF analyzed the data and wrote the manuscript.

## Funding

This research was supported by an A-Base research project of Agriculture and Agri-Food Canada to Y-BF, and an NSERC Discovery grant to HW.

## Conflict of Interest

The authors declare that the research was conducted in the absence of any commercial or financial relationships that could be construed as a potential conflict of interest.
