# Supplementary figures and images for: Arabidopsis Seed Stored mRNAs are Degraded Constantly over Aging Time, as Revealed by New Quantification Methods
Source: Front Plant Sci. 2020 Jan 29;10:1764. doi: 10.3389/fpls.2019.01764 (PMC7000544; doi:10.3389/fpls.2019.01764)

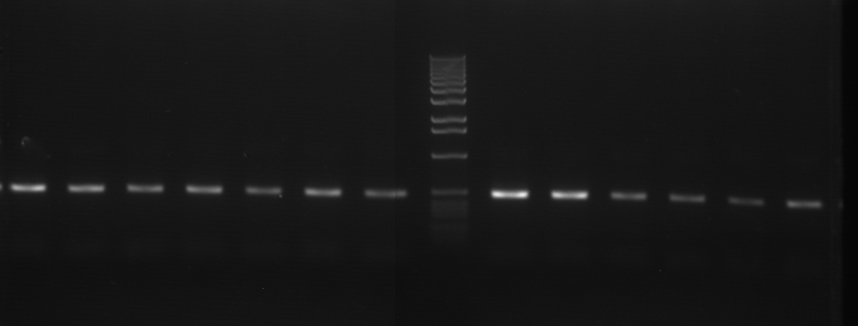

Supplement: Supplementary file 1 [file DataSheet_1.zip › Figure 1_Original Image_190730/Group A/A1.tif]

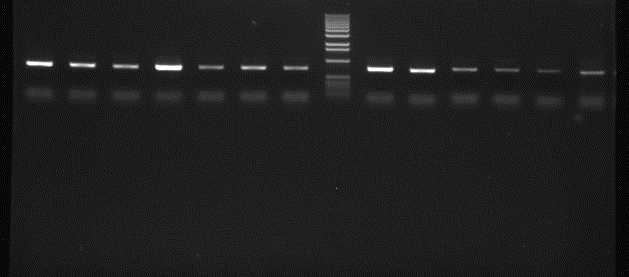

Supplement: Supplementary file 1 [file DataSheet_1.zip › Figure 1_Original Image_190730/Group A/A10.TIF]

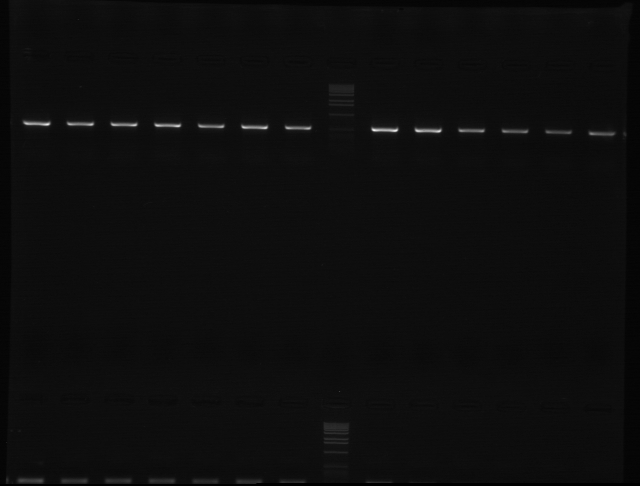

Supplement: Supplementary file 1 [file DataSheet_1.zip › Figure 1_Original Image_190730/Group A/A13.TIF]

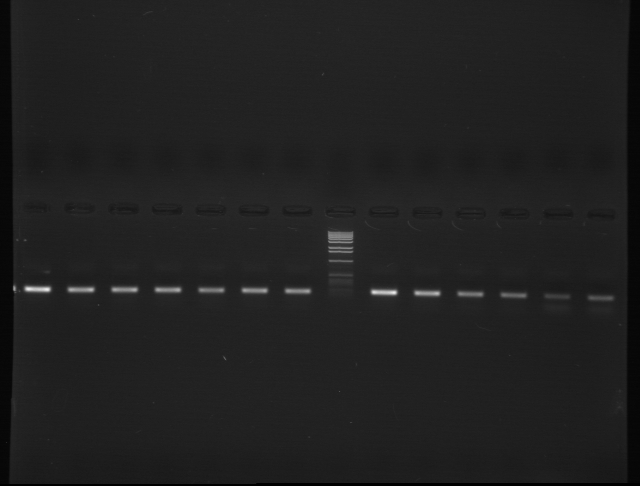

Supplement: Supplementary file 1 [file DataSheet_1.zip › Figure 1_Original Image_190730/Group A/A14.TIF]

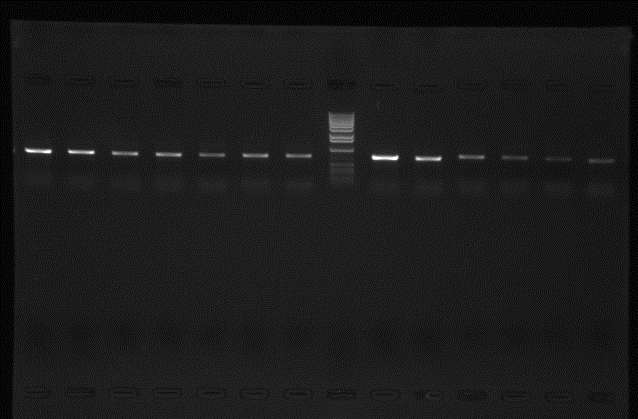

Supplement: Supplementary file 1 [file DataSheet_1.zip › Figure 1_Original Image_190730/Group A/A15.TIF]

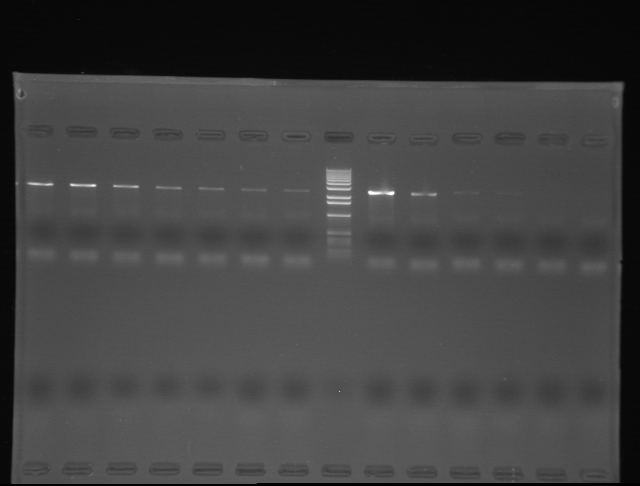

Supplement: Supplementary file 1 [file DataSheet_1.zip › Figure 1_Original Image_190730/Group A/A16.TIF]

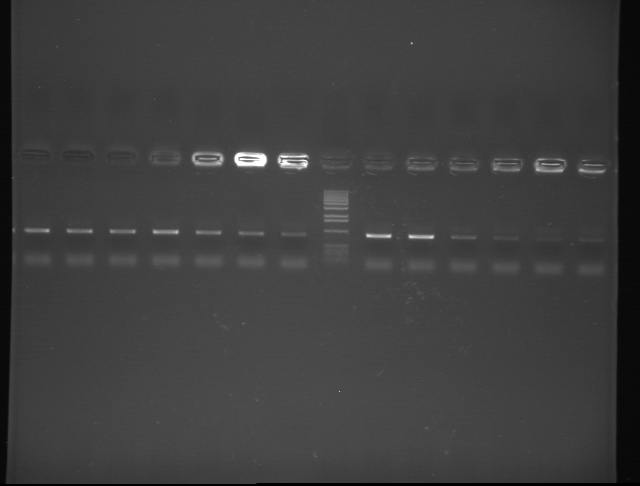

Supplement: Supplementary file 1 [file DataSheet_1.zip › Figure 1_Original Image_190730/Group A/A17.TIF]

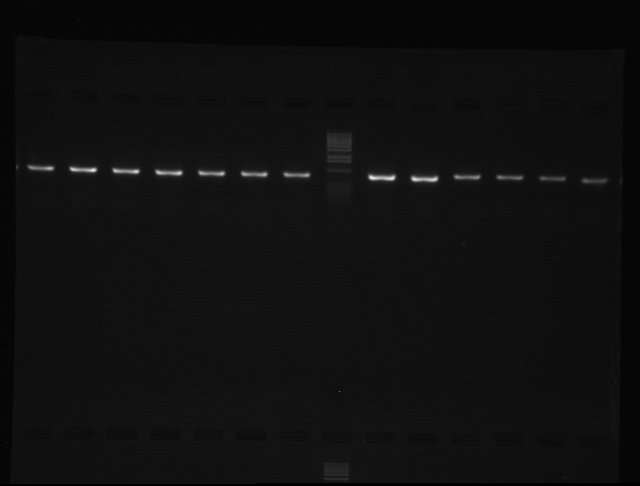

Supplement: Supplementary file 1 [file DataSheet_1.zip › Figure 1_Original Image_190730/Group A/A18.TIF]

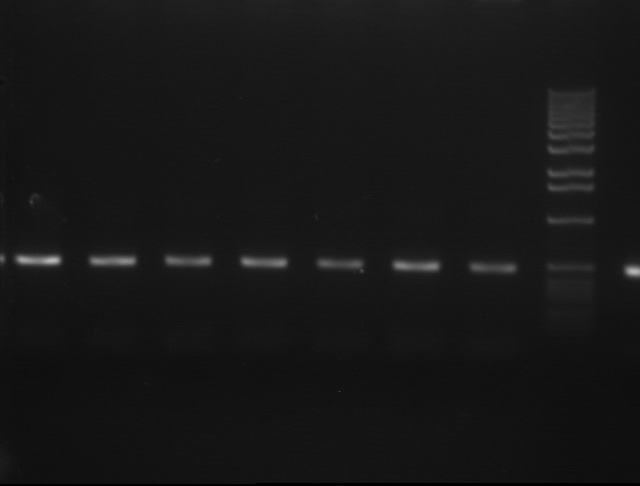

Supplement: Supplementary file 1 [file DataSheet_1.zip › Figure 1_Original Image_190730/Group A/A1a NA seeds.TIF]

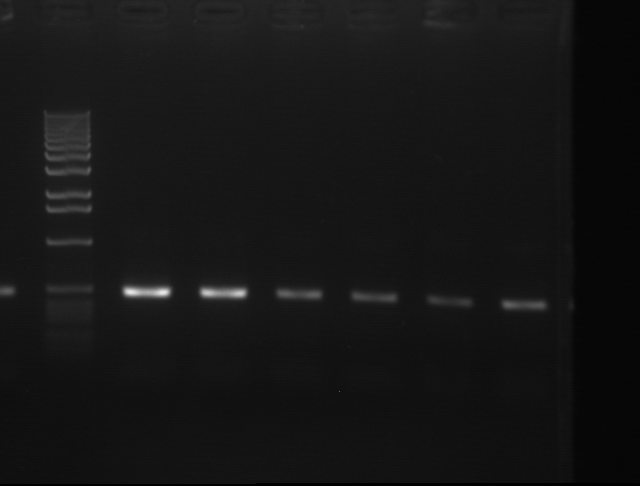

Supplement: Supplementary file 1 [file DataSheet_1.zip › Figure 1_Original Image_190730/Group A/A1b AA seeds.TIF]

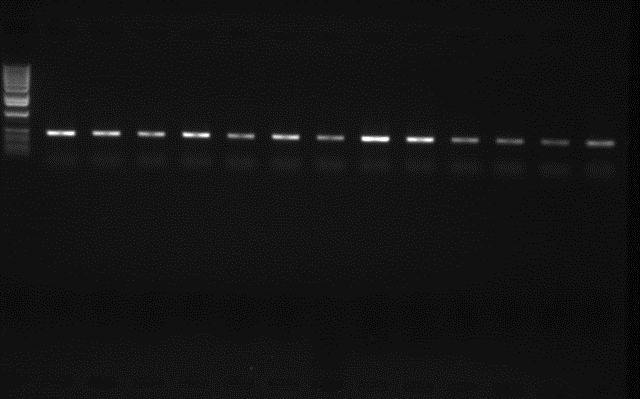

Supplement: Supplementary file 1 [file DataSheet_1.zip › Figure 1_Original Image_190730/Group A/A2.TIF]

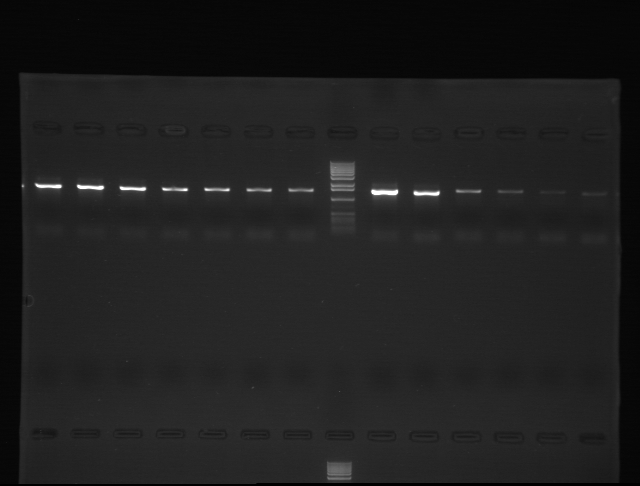

Supplement: Supplementary file 1 [file DataSheet_1.zip › Figure 1_Original Image_190730/Group A/A20.TIF]

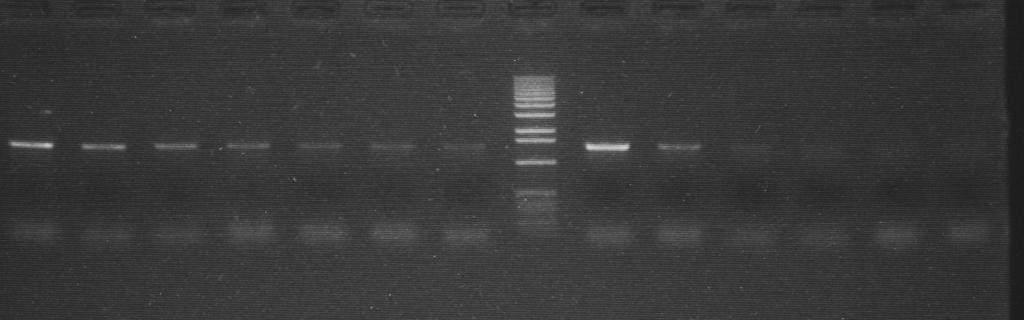

Supplement: Supplementary file 1 [file DataSheet_1.zip › Figure 1_Original Image_190730/Group A/A21.jpg]

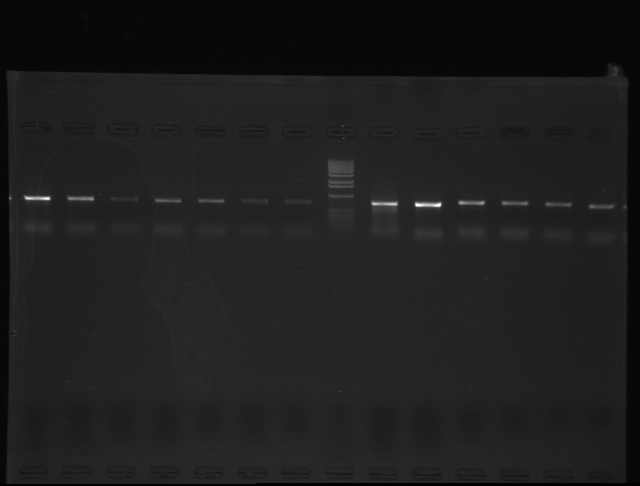

Supplement: Supplementary file 1 [file DataSheet_1.zip › Figure 1_Original Image_190730/Group A/A22.TIF]

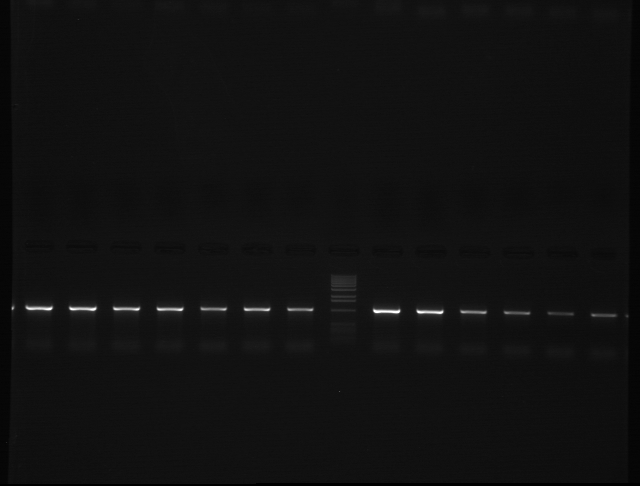

Supplement: Supplementary file 1 [file DataSheet_1.zip › Figure 1_Original Image_190730/Group A/A23.TIF]

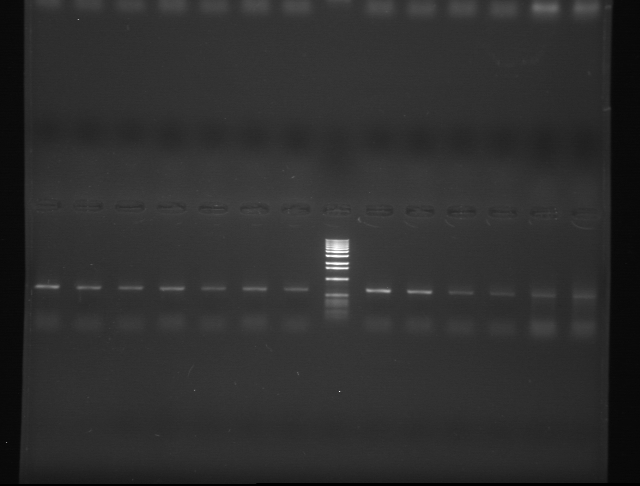

Supplement: Supplementary file 1 [file DataSheet_1.zip › Figure 1_Original Image_190730/Group A/A26.TIF]

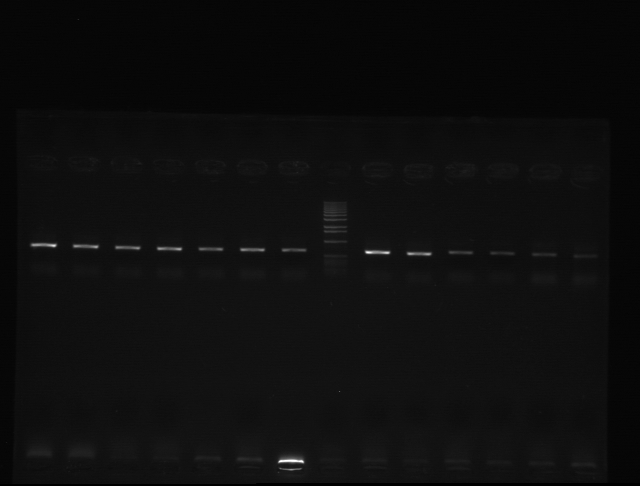

Supplement: Supplementary file 1 [file DataSheet_1.zip › Figure 1_Original Image_190730/Group A/A27.TIF]

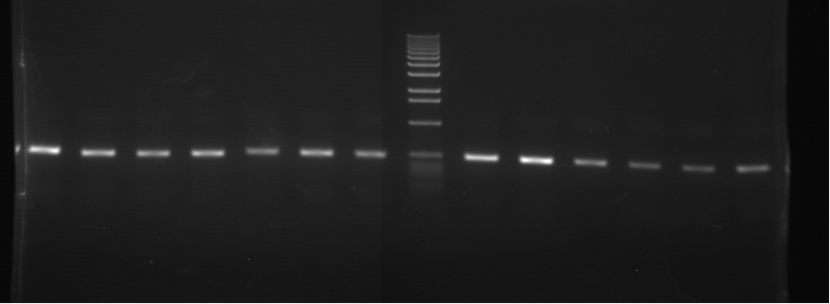

Supplement: Supplementary file 1 [file DataSheet_1.zip › Figure 1_Original Image_190730/Group A/A3.tif]

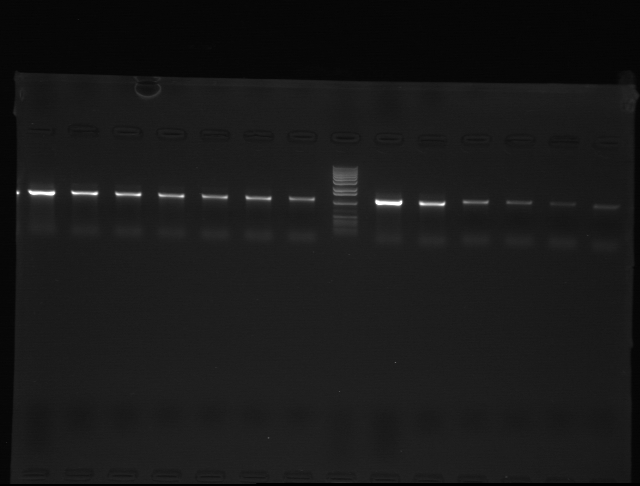

Supplement: Supplementary file 1 [file DataSheet_1.zip › Figure 1_Original Image_190730/Group A/A30.TIF]

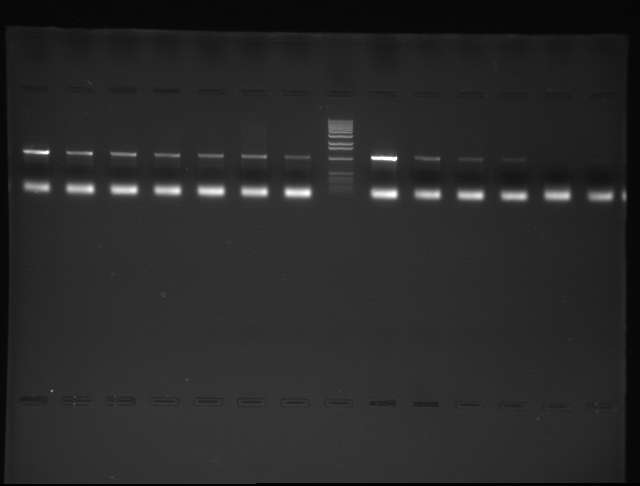

Supplement: Supplementary file 1 [file DataSheet_1.zip › Figure 1_Original Image_190730/Group A/A31.TIF]

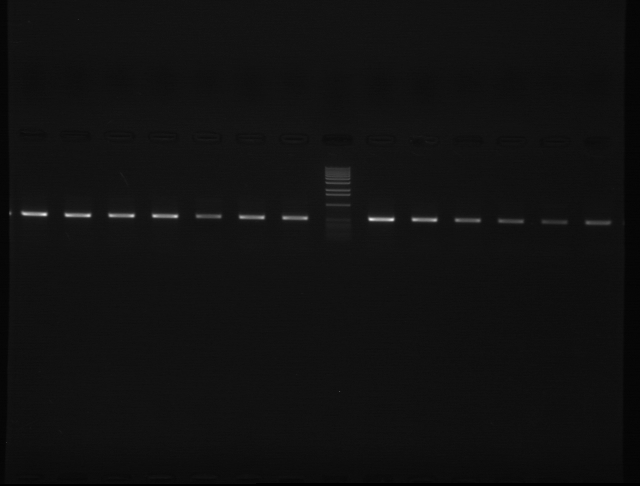

Supplement: Supplementary file 1 [file DataSheet_1.zip › Figure 1_Original Image_190730/Group A/A33.TIF]

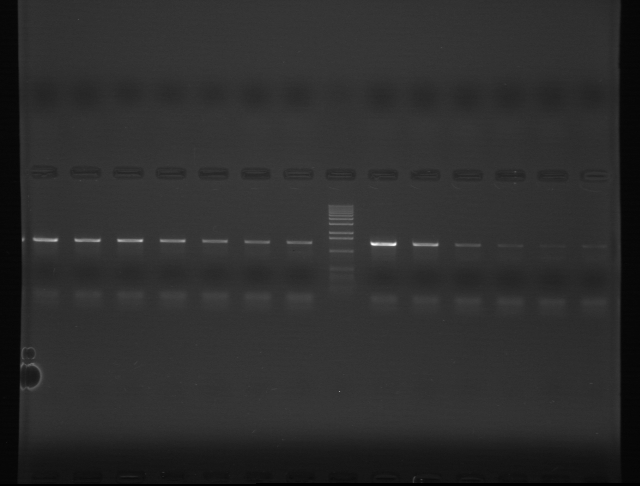

Supplement: Supplementary file 1 [file DataSheet_1.zip › Figure 1_Original Image_190730/Group A/A35.TIF]

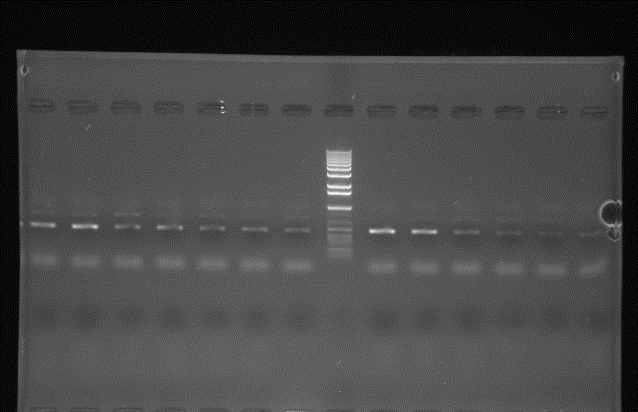

Supplement: Supplementary file 1 [file DataSheet_1.zip › Figure 1_Original Image_190730/Group A/A37.TIF]

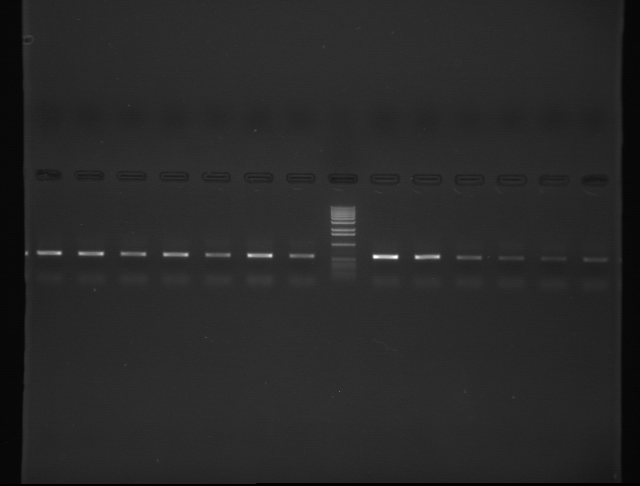

Supplement: Supplementary file 1 [file DataSheet_1.zip › Figure 1_Original Image_190730/Group A/A38.TIF]

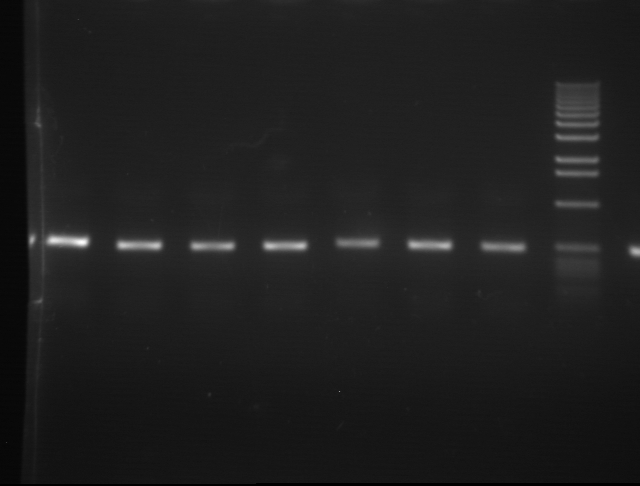

Supplement: Supplementary file 1 [file DataSheet_1.zip › Figure 1_Original Image_190730/Group A/A3a NA seeds.TIF]

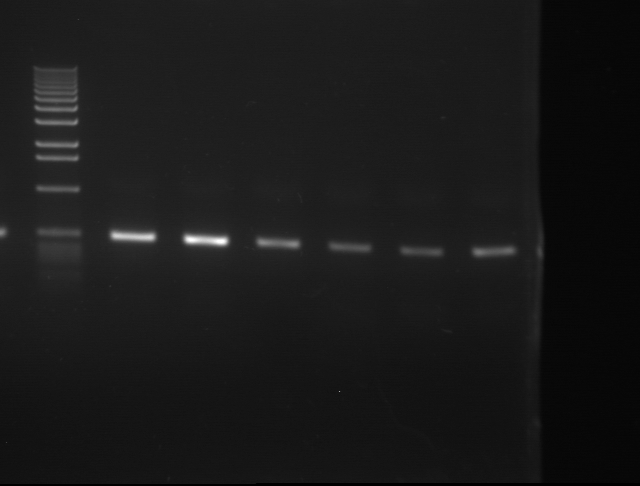

Supplement: Supplementary file 1 [file DataSheet_1.zip › Figure 1_Original Image_190730/Group A/A3b AA seeds.TIF]

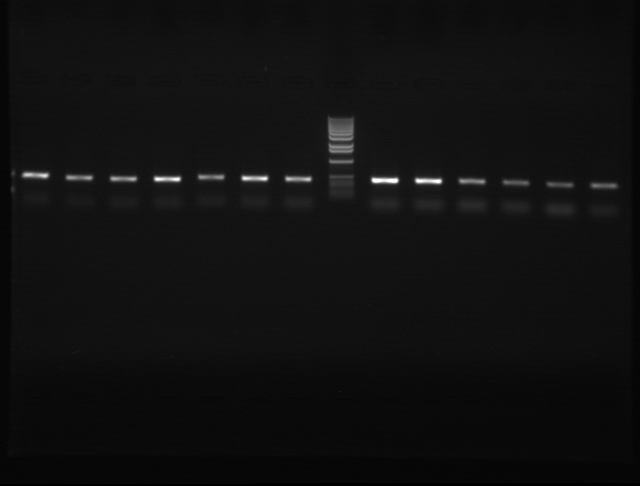

Supplement: Supplementary file 1 [file DataSheet_1.zip › Figure 1_Original Image_190730/Group A/A4.TIF]

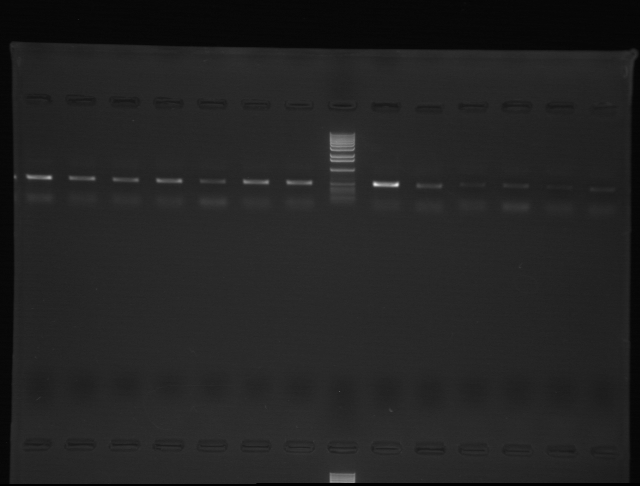

Supplement: Supplementary file 1 [file DataSheet_1.zip › Figure 1_Original Image_190730/Group A/A40.TIF]

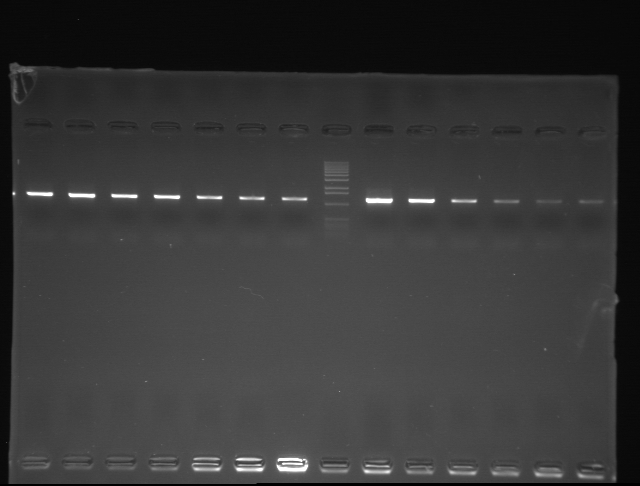

Supplement: Supplementary file 1 [file DataSheet_1.zip › Figure 1_Original Image_190730/Group A/A41.TIF]

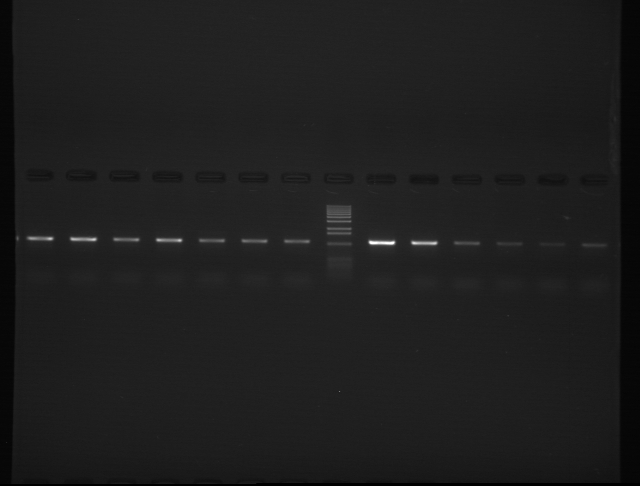

Supplement: Supplementary file 1 [file DataSheet_1.zip › Figure 1_Original Image_190730/Group A/A42.TIF]

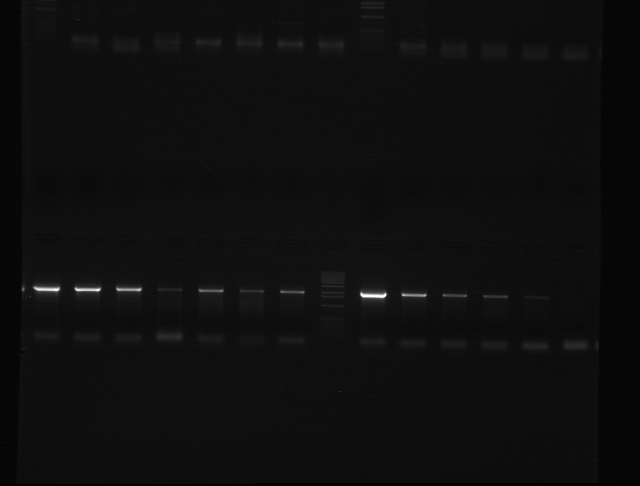

Supplement: Supplementary file 1 [file DataSheet_1.zip › Figure 1_Original Image_190730/Group A/A44.TIF]

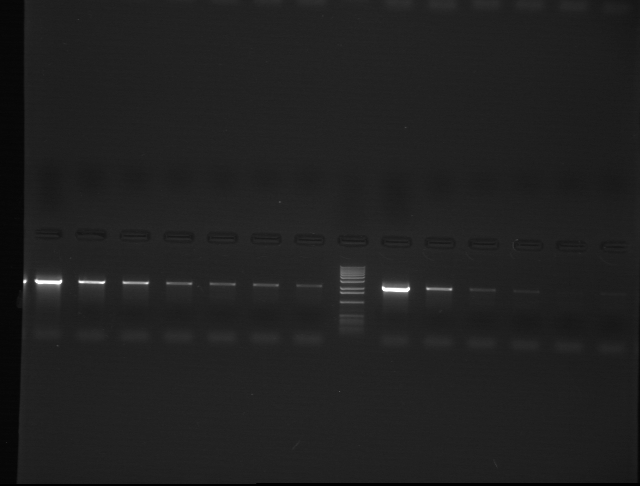

Supplement: Supplementary file 1 [file DataSheet_1.zip › Figure 1_Original Image_190730/Group A/A45.TIF]

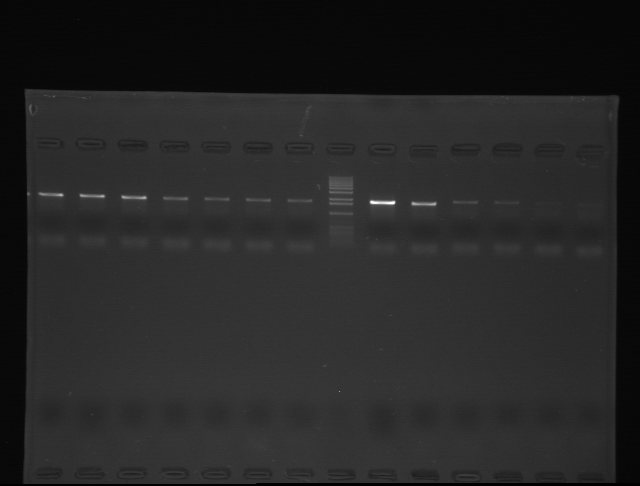

Supplement: Supplementary file 1 [file DataSheet_1.zip › Figure 1_Original Image_190730/Group A/A47.TIF]

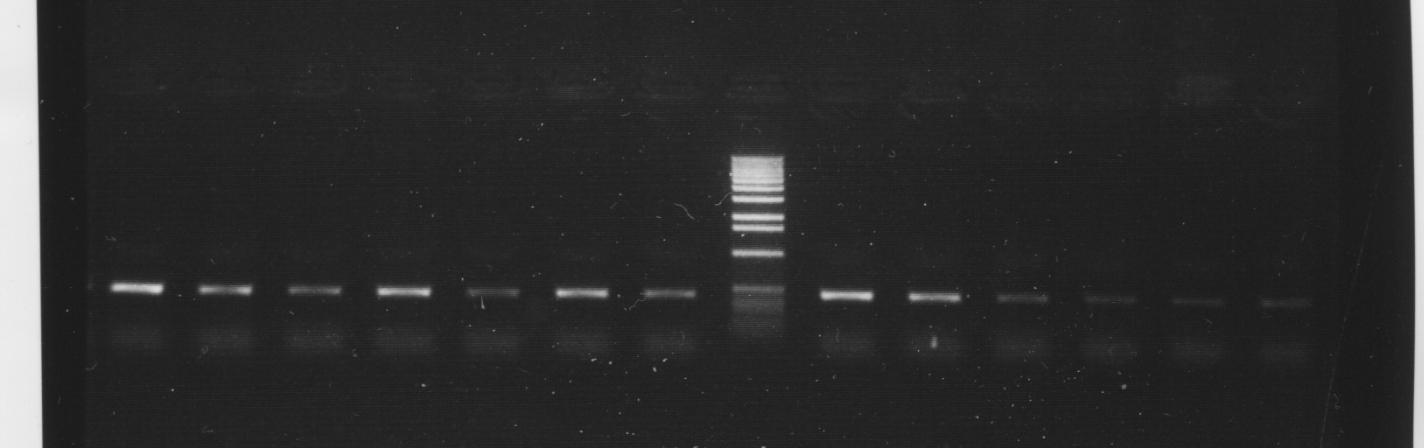

Supplement: Supplementary file 1 [file DataSheet_1.zip › Figure 1_Original Image_190730/Group A/A5.jpg]

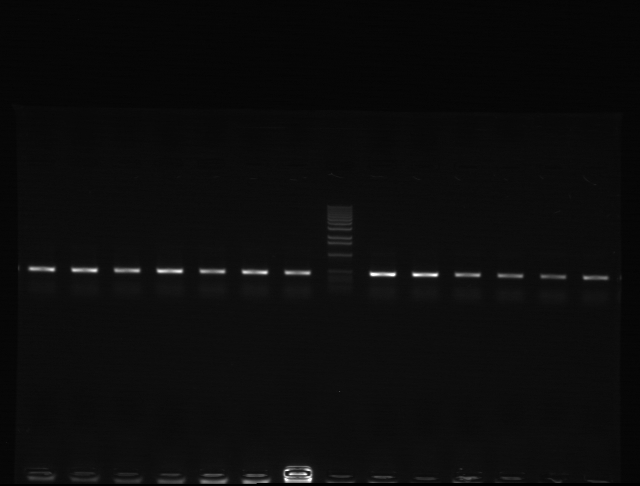

Supplement: Supplementary file 1 [file DataSheet_1.zip › Figure 1_Original Image_190730/Group A/A6.TIF]

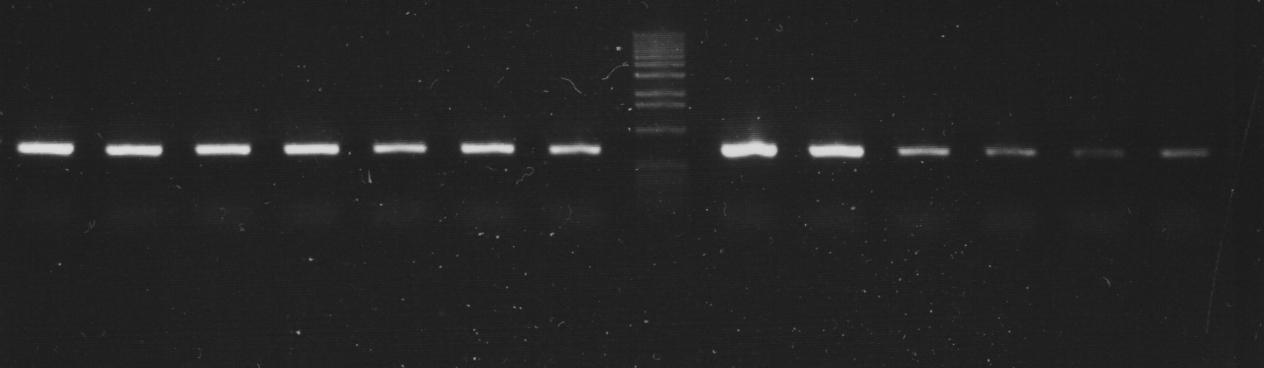

Supplement: Supplementary file 1 [file DataSheet_1.zip › Figure 1_Original Image_190730/Group A/A7.jpg]

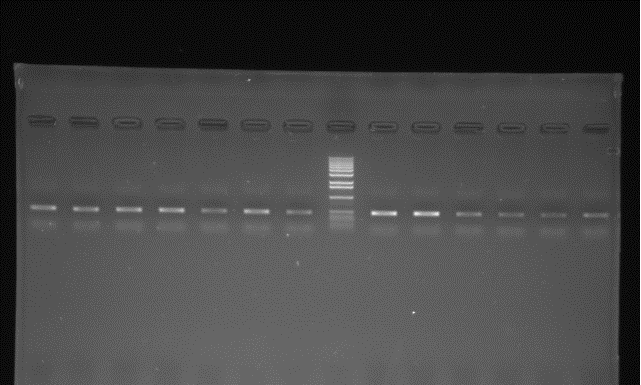

Supplement: Supplementary file 1 [file DataSheet_1.zip › Figure 1_Original Image_190730/Group A/A8.TIF]

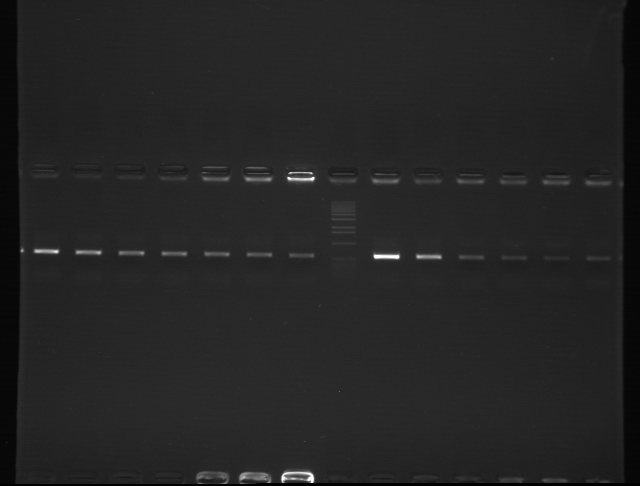

Supplement: Supplementary file 1 [file DataSheet_1.zip › Figure 1_Original Image_190730/Group B/B1.TIF]

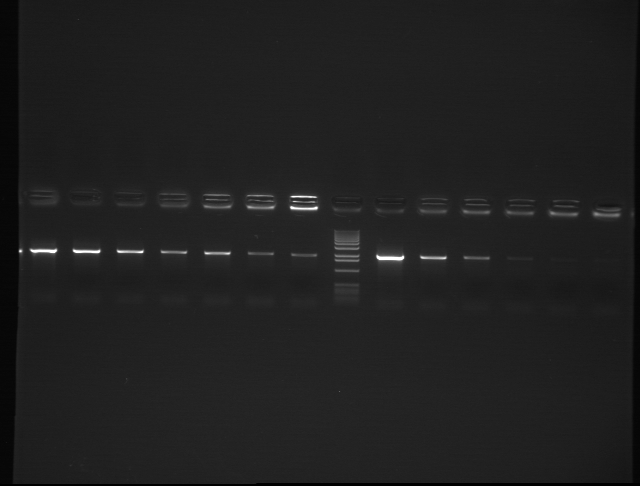

Supplement: Supplementary file 1 [file DataSheet_1.zip › Figure 1_Original Image_190730/Group B/B10.TIF]

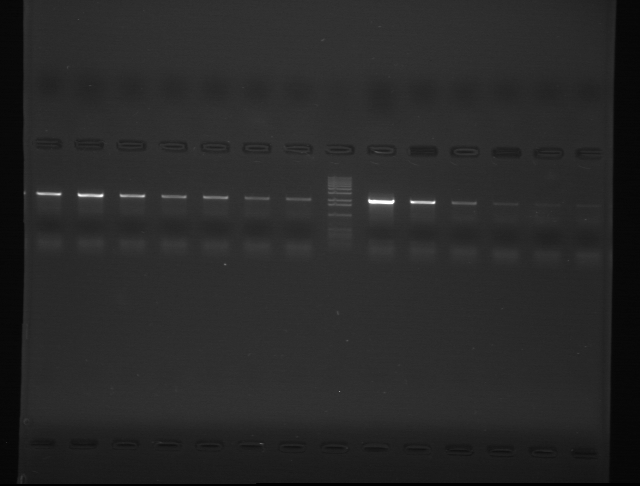

Supplement: Supplementary file 1 [file DataSheet_1.zip › Figure 1_Original Image_190730/Group B/B12.TIF]

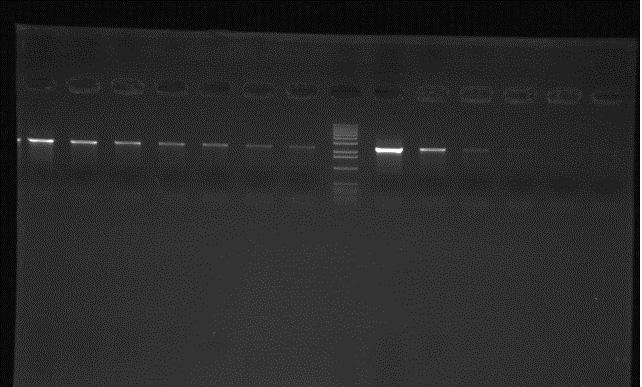

Supplement: Supplementary file 1 [file DataSheet_1.zip › Figure 1_Original Image_190730/Group B/B13.TIF]

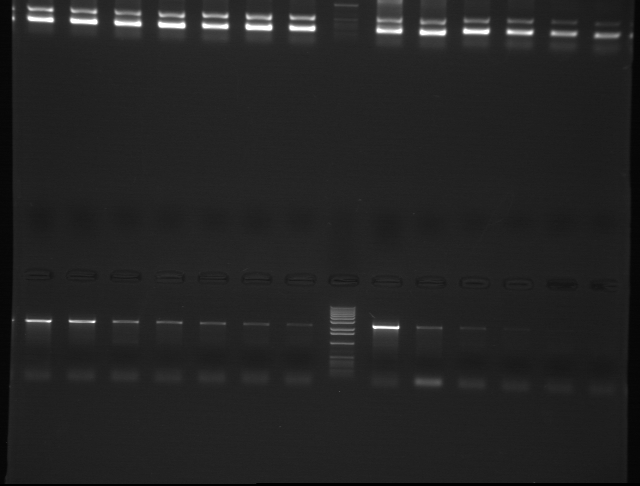

Supplement: Supplementary file 1 [file DataSheet_1.zip › Figure 1_Original Image_190730/Group B/B14.TIF]

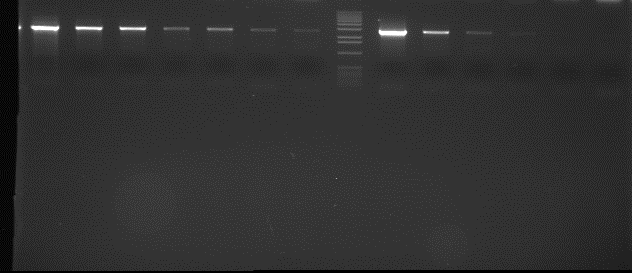

Supplement: Supplementary file 1 [file DataSheet_1.zip › Figure 1_Original Image_190730/Group B/B16.TIF]

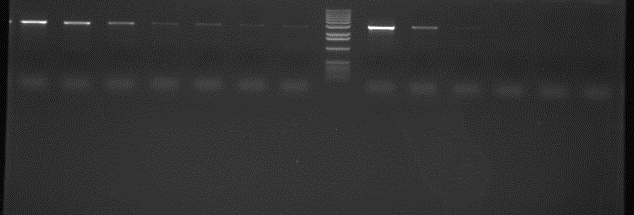

Supplement: Supplementary file 1 [file DataSheet_1.zip › Figure 1_Original Image_190730/Group B/B17.TIF]

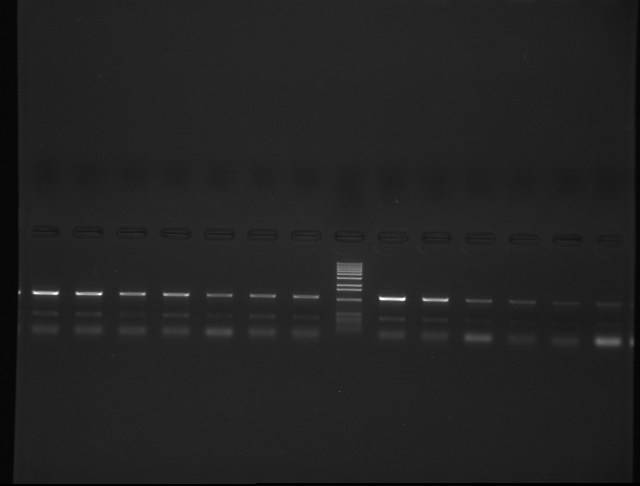

Supplement: Supplementary file 1 [file DataSheet_1.zip › Figure 1_Original Image_190730/Group B/B2.TIF]

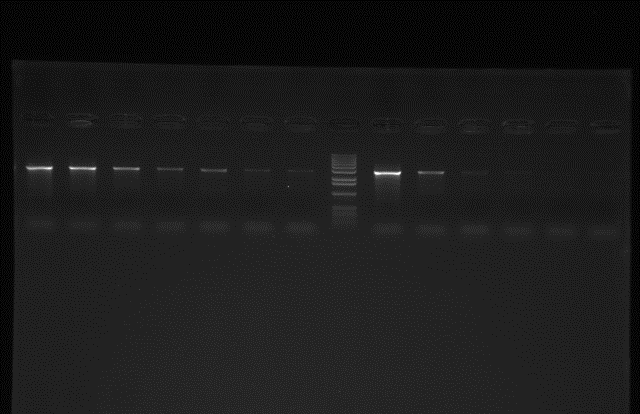

Supplement: Supplementary file 1 [file DataSheet_1.zip › Figure 1_Original Image_190730/Group B/B20.TIF]

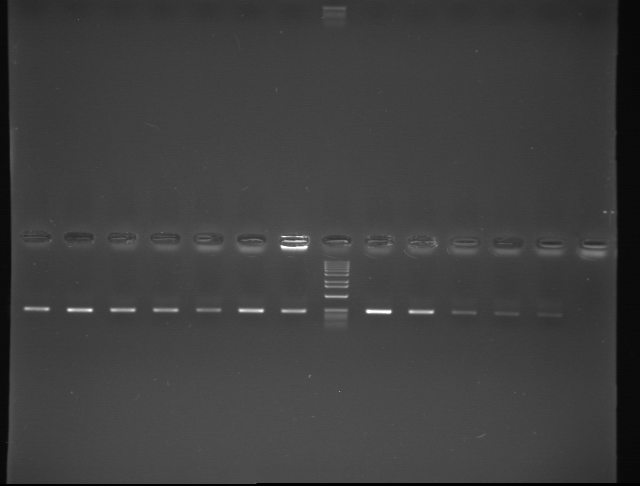

Supplement: Supplementary file 1 [file DataSheet_1.zip › Figure 1_Original Image_190730/Group B/B3.TIF]

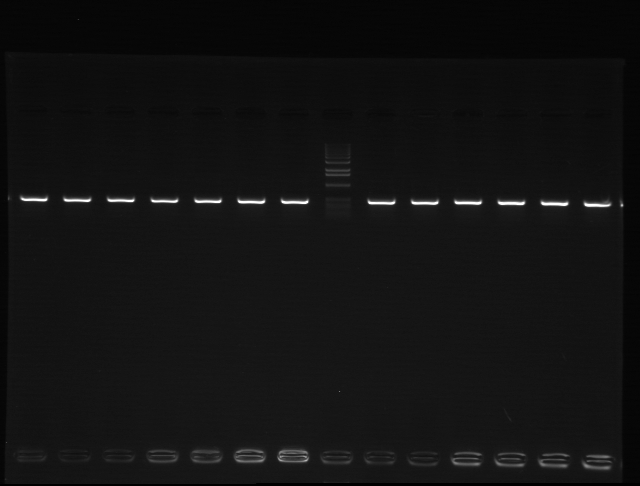

Supplement: Supplementary file 1 [file DataSheet_1.zip › Figure 1_Original Image_190730/Group B/B4.TIF]

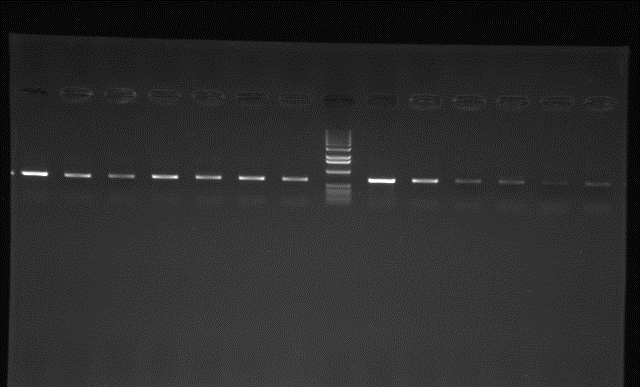

Supplement: Supplementary file 1 [file DataSheet_1.zip › Figure 1_Original Image_190730/Group B/B5.TIF]

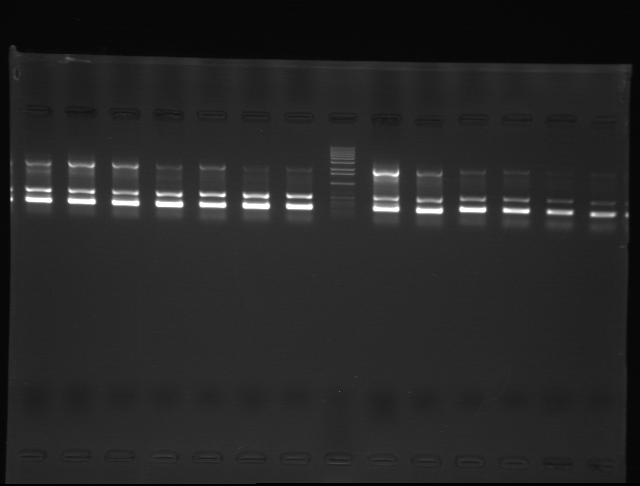

Supplement: Supplementary file 1 [file DataSheet_1.zip › Figure 1_Original Image_190730/Group B/B6.TIF]

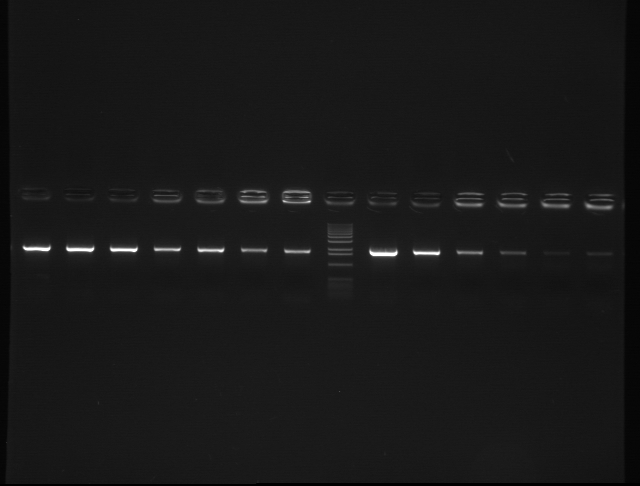

Supplement: Supplementary file 1 [file DataSheet_1.zip › Figure 1_Original Image_190730/Group B/B7.TIF]

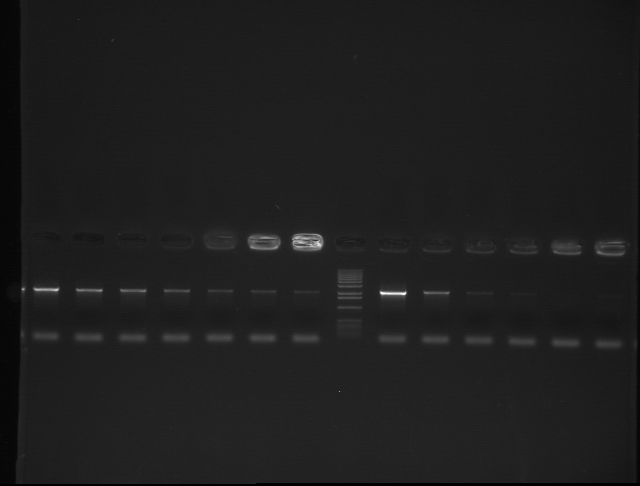

Supplement: Supplementary file 1 [file DataSheet_1.zip › Figure 1_Original Image_190730/Group B/B8.TIF]

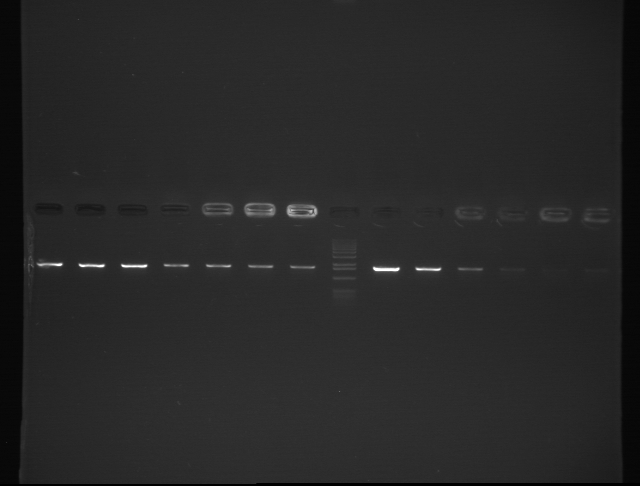

Supplement: Supplementary file 1 [file DataSheet_1.zip › Figure 1_Original Image_190730/Group B/B9.TIF]
