# Supplementary material for: Arabidopsis Seed Stored mRNAs are Degraded Constantly over Aging Time, as Revealed by New Quantification Methods
Source: Front Plant Sci. 2020 Jan 29;10:1764. doi: 10.3389/fpls.2019.01764 (PMC7000544; doi:10.3389/fpls.2019.01764)
Supplement: Supplementary file 2 [file DataSheet_2.pdf]

SUPPLEMENTARY MATERIAL

Table S1. List of naturally aged Arabidopsis seeds used in this study.

Harvest time	Germination percentage ± SE
Sep.2014	100.0 ± 0.0
Mar.2010	99.0 ± 0.0
Sep.2003	97.3 ± 2.5
Dec.1999	68.3 ± 0.6
Dec.1997	45.0 ± 2.0
Dec.1997	10.0 ± 1.1
Dec.1997	2.0 ± 0.3
Dec.1997	0.5 ± 0.5

Arabidopsis seeds were collected from mature plants, placed in 2.0 mL tubes with a screw cap, and stored in a 4ºC refrigerator. For germination assay, seeds were sterilized and spot-placed on ½ MS plates containing 1% sucrose. Each seed lot had at least three plates with each plate having 100 seeds. Plates were placed in a tissue culture chamber, and after 7 days surveyed for seed germination. The germination percentage with standard error (SE) is shown. The germination assay was performed in Mar. 2015.

Table S2. **List of genes used to survey the presence of seed stored mRNAs by RT-PCR**.

Protein ubiquitination (group A)			Heat shock proteins (group B)			Cell cycle and growth (group C)			Others (group D)		
**Gene code**	**Gene ID**	**CDS (bps)**	**Gene code**	**Gene ID**	**CDS (bps)**	**Gene code**	**Gene ID**	**CDS (bps)**	**Gene code**	**Gene ID**	**CDS (bps)**
A1	At1g78870	441	B1	At4g10250	590	C1	At3g48750	885	D1	At3g62060	1260
A2	At1g16890	489	B2	At5g59720	490	C2	At5g10270	1518	D2	At2g46930	1251
A3	At1g23260	477	B3	At1g59860	460	C3	At1g73690	1197	D3	At3g16090	1479
A4	At2g36060	441	B4	At5g12030	461	C4	At1g44110	1383	D4	At5g27150	1617
A5	At5g41700	450	B5	At4g27670	685	C5	At1g77390	1329	D5	At1g15690	2313
A6	At5g56150	447	B6	At5g02490	1962	C6	At5g43080	1068	D6	At2g01980	3441
A7	At1g17280	714	B7	At1g16030	1940	C7	At2g17620	1290	D7	At2g39800	2154
A8	At5g62540	453	B8	At4g24280	2160	C8	At1g16330	1947	D8	At1g28120	921
A9	At5g59300	612	B9	At2g32120	1692	C9	At1g70210	1020	D9	At1g18860	1443
A10	At5g05080	756	B10	At5g09590	2049	C10	At2g22490	1089	D10	At1g13930	468
A11	At1g36340	465	B11	At5g52640	2118	C11	At4g34160	1131	D11	At2g39730	1425
A12	At3g24515	1230	B12	At5g56000	2100	C12	At5g65420	957	D12	At4g20360	1431
A13	At5g41340	561	B13	At2g04030	2343	C13	At4g37630	972	D13	At2g30870	648
A14	At1g63800	558	B14	At3g07770	2400	C14	At4g03270	909	D14	At3g26650	1191
A15	At2g46030	552	B15	At4g24190	2472	C15	At2g23430	576	D15	At3g26650	1737
A16	At5g33393	1308	B16	At1g74310	2736	C16	At3g50630	630	D16	At2g43030	816
A17	At5g33393	837	B17	At5g15450	2907	C17	At3g24810	570	D17	At5g11670	1767
A18	At1g74370	786	B18	At2g25140	2895	C18	At3g19150	591	D18	At1g54870	1008
A19	At4g14220	1116	B19	At3g48870	2859	C19	At1g49620	588	D19	At5g03860	1689
A20	At3g01650	1470	B20	At5g51070	2838	C20	At5g48820	723	D20	At3g08030	1098
A21	At5g14420	1407				C21	At2g32710	870			
A22	At2g42360	711				C22	At3g12280	3042			
A23	At2g47700	1077				C23	At1g59580	1131			
A24	At4g11680	1173				C24	At1g07370	1025			
A25	At5g37270	627				C25	At2g29570	795			
A26	At5g38895	981				C26	At1g04820	1353			
A27	At5g41350	639				C27	At5g23860	1350			
A28	At1g73760	1104				C28	At3g16640	507			
A29	At3g58720	801				C29	At1g19850	2709			
A30	At5g08139	1131				C30	At1g30330	2808			
A31	At1g55530	1056				C31	At1g59750	1998			
A32	At3g20395	672				C32	At1g19220	3261			
A33	At2g17730	762									
A34	At5g73270	627									
A35	At5g47570	378									
A36	At5g05130	2589									
A37	At4g23450	627									
A38	At4g00335	573									
A39	At3g16090	1479									
A40	At1g63170	1146									
A41	At1g12760	1227									
A42	At3g61180	1140									
A43	At1G23030	1839									
A44	At3G46510	1983									
A45	At1G29340	2190									
A46	At2g35930	1236									
A47	At5G67530	1788									
A48	At5G61560	2391									

A total of 120 genes were tested and they are loosely grouped into four groups: 48 genes in protein ubiquitination group (group A), 20 genes in heat shock protein group (group B), 32 genes in cell cycle and growth group (group C), and 20 other genes in the last group (group D). For convenience of referring in this study, each gene is given a gene code. The gene ID and the length of cDNA coding sequence (CDS) in basepairs (bps) are indicated.

Table S3. △Ct values for 29 genes in qPCR analysis on changes in stored mRNAs in aged seeds.

Gene code	Gene ID	Size (bps)	Average Ct value ± standard error			△Ct	
			Control	AA	NA	AA	NA
A18	At1g74370	786	20.9±0.2	22.1±0.0	21.9±0.1	1.1±0.1	0.9±0.1
A20	At3g01650	1470	22.1±0.1	23.6±0.1	24.2±0.2	1.6±0.1	2.2±0.2
A21	At5g14420	1407	24.1±0.2	25.6±0.1	24.8±0.2	1.5±0.1	0.7±0.2
A26	At5g38895	981	22.0±0.1	23.2±0.6	23.8±0.3	1.2±0.4	1.8±0.1
A27	At5g41350	639	21.4±0.3	22.6±0.1	22.1±0.2	1.2±0.1	0.7±0.2
A31	At1g55530	1056	23.9±0.1	25.6±0.3	24.8±0.2	1.7±0.3	0.8±0.2
A33	At2g17730	762	27.0±0.0	28.4±0.1	27.8±0.1	1.4±0.1	0.8±0.1
A35	At5g47570	1400	26.8±0.1	28.5±0.1	27.9±0.1	1.6±0.1	1.0±0.1
A38	At4g00335	537	25.8±0.2	26.3±0.1	25.8±0.2	0.6±0.1	0.1±0.2
A40	At1g63170	1146	27.3±0.1	28.5±0.1	28.0±0.1	1.2±0.1	0.7±0.1
A41	At1g12760	1227	24.2±0.1	26.8±0.2	25.6±0.2	2.6±0.2	1.4±0.2
A42	At3g61180	1140	26.0±0.0	27.8±0.2	26.9±0.2	1.8±0.2	0.9±0.2
A44	At3G46510	1983	26.2±0.0	27.4±0.3	28.1±0.1	1.3±0.3	1.9±0.1
A45	At1g29340	2190	25.1±0.2	27.3±0.1	27.5±0.1	2.2±0.1	2.4±0.1
A47	At5G67530	1788	28.2±0.3	31.5±0.2	29.7±0.1	3.3±0.2	1.5±0.1
B1	At4g10250	590	24.5±0.3	25.4±0.1	25.4±0.1	0.9±0.1	0.9±0.1
B2	At5g59720	490	27.9±0.5	28.5±0.5	28.4±0.1	0.5±0.5	0.5±0.1
B4	At5g12030	461	15.6±0.0	16.3±0.0	15.9±0.1	0.7±0.0	0.4±0.1
B5	At4g27670	685	28.3±0.3	28.8±0.0	27.3±0.1	0.5±0.0	0.9±0.1
B7	At1g16030	1940	18.8±0.2	23.5±0.2	22.7±0.2	4.7±0.2	3.9±0.2
B9	At2g32120	1692	22.1±0.1	25.6±0.1	24.5±0.2	3.5±0.1	2.3±0.2
B10	At5g09590	2049	24.9±0.2	29.2±0.1	28.0±0.1	4.2±0.0	3.1±0.1
B12	At5g56000	2100	26.8±0.2	30.6±0.5	29.7±0.2	3.8±0.5	2.9±0.2
B13	At2g04030	2343	26.6±0.3	31.4±0.2	30.4±0.3	4.8±0.2	3.8±0.3
B14	At3g07770	2400	28.8±0.2	31.5±0.1	31.9±0.4	2.6±0.1	3.1±0.4
B16	At1g74310	2736	23.2±0.3	29.9±0.1	25.8±0.2	6.7±0.2	2.5±0.1
B17	At5g15450	2907	29.5±0.1	32.8±0.2	32.2±0.1	3.3±0.2	2.7±0.1
B20	At5g51070	2838	25.3±0.3	32.2±0.2	31.0±0.1	6.9±0.2	5.8±0.4
D5	At1g15690	2313	23.7±0.2	28.4±0.3	27.4±0.2	4.7±0.3	3.7±0.2

cDNAs of control (100% germination), artificially aged (AA, 0.5% germination) and naturally aged (NA, 0.5% germination) Arabidopsis seeds were used. All Ct values are the average of three biological repeats. For each gene analyzed, the △Ct = Ct of aged sample – Ct of control.

Table S4. List of cDNA fragments of six genes and the primers used in qPCR analysis.

Fragment code	Fragment Length (bps)	Forward primer	Reverse primer
$B16_{250}$	250	AGAACTGTCAAAGATGGTTGTG	TTAATCCTCGATCATTTCCTCA
$B16_{500}$	500	AGGTGAGGAAACACTTCAGAC	TTAATCCTCGATCATTTCCTCA
$B16_{1000}$	1000	TGCAGTTTCTGAGGCAATTCT	TTAATCCTCGATCATTTCCTCA
$B16_{1500}$	1500	ATTGATAACCTTGAAAGGAAGAG	TTAATCCTCGATCATTTCCTCA
$B16_{2000}$	2000	TAGTTGCTGGTGCTAAATACC	TTAATCCTCGATCATTTCCTCA
$B16_{2500}$	2500	ACCTGATGATATTCCAGCGAG	TTAATCCTCGATCATTTCCTCA
$B20_{250}$	250	TTTAGAGGTGTCTGAACCCGT	CTATGCGATCGATGTTTTGTC
$B20_{500}$	500	GATCATTGGCCATTGCAAAGG	CTATGCGATCGATGTTTTGTC
$B20_{1000}$	1000	TGAAAGAATGCTTCTTATGAGTC	CTATGCGATCGATGTTTTGTC
$B20_{1500}$	1500	TTTCAGCCAGTGTTGATTAACG	CTATGCGATCGATGTTTTGTC
$B20_{2000}$	2000	CACGTGCTAGTGAGGGTCTT	CTATGCGATCGATGTTTTGTC
$B14_{2000}$	2000	ATAAGCTTCGTTATTTGAGCGT	TCATTTCTTCCCATCCACTTC
$C22_{2000}$	2000	ATAGCTTACTTGGATCTGGAAG	CTATGAATCTGTTGGCTCGG
$B10_{2000}$	2000	CCTCTCCTTTCTCAGCTTAC	TCACTTTTTCACTTCCTCGTACT
$D5_{2000}$	2000	GAAAAGGATACCACCGTGAGT	GTGTCTTCATGATTTTCTTTGC

The fragment length (in basepairs, bps) starts from the STOP codon counting towards the 5'-direction of a stored mRNA. Primer sequences are listed from the 5' end to 3' end.

Table S5. **The 25S/18S ratios and RNA integrity numbers (RINs) of RNA sample isolated from control and aged Arabidopsis seeds.**

Germination Percentage (%)		NA							AA					
		100	99.0	87.3	68.3	46.3	10	0	100	92.7	52.0	20.7	3.7	0
25S/18S	Repeat 1	1.73	1.75	1.68	1.58	2.63	1.85	1.66	1.81	1.62	1.83	2.14	1.63	1.79
	Repeat 2	1.67	1.71	1.5	1.25	2.05	1.5	2.08	1.54	1.7	1.74	1.45	1.78	1.91
RIN	Repeat 1	8.3	8.8	8.5	8.5	8.4	8.8	7.2	9.4	8.5	8.5	8.6	8.6	8.7
	Repeat 2	8.3	9.2	8.2	8	8.4	8	8.2	8.2	8.3	8.3	8.1	8.6	8.8

Total RNAs were isolated from naturally aged (NA) and artificially aged (AA) as well as unaged control Arabidopsis seeds. For each aging point, two samples were used for RNA analysis by Agilent 2100 Bioanalyzer to obtain the 25S/18S ratio and RIN.

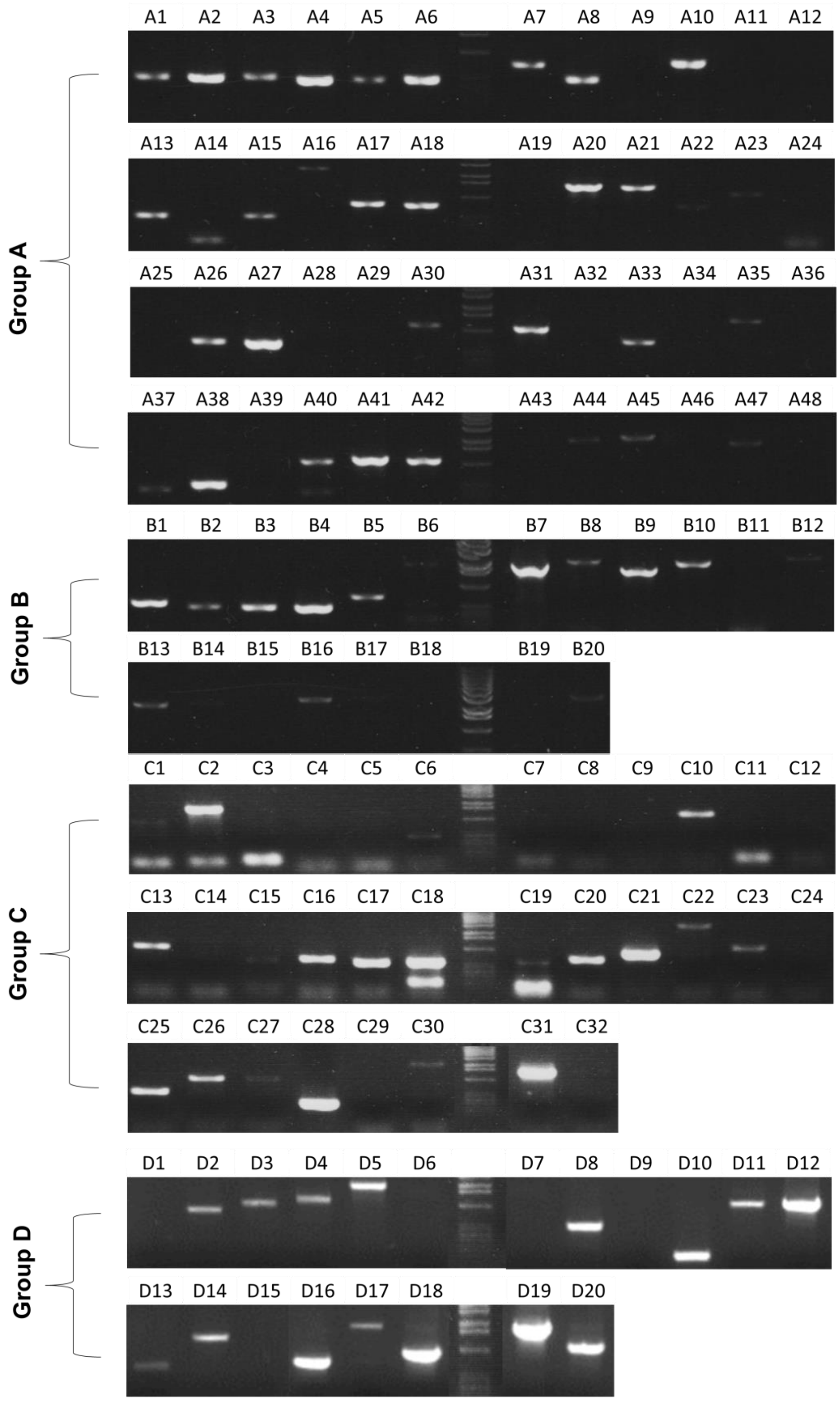

Figure S1. **Results of RT-PCR analysis of 120 genes (in A, B, C and D groups) to survey the presence of stored mRNAs in Arabidopsis dry seeds**. Information on these genes is provided in Table S2. cDNA reverse-transcribed from total RNA of unaged dry seeds was used in PCR with primers specific for each of the genes. The PCR products were subjected to electrophoresis in 1% agarose gel.

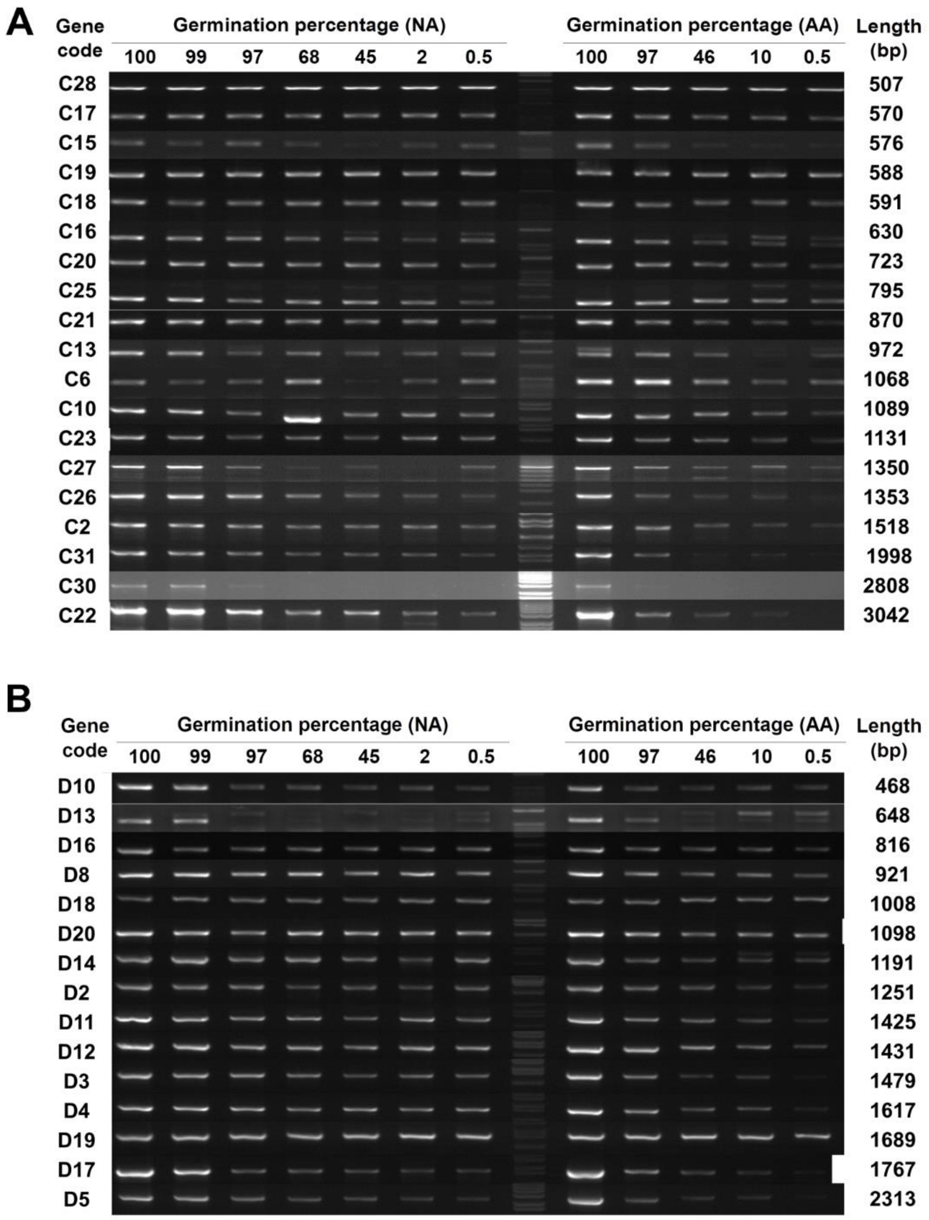

Figure S2. The level of stored mRNAs in naturally aged (NA) and artificially aged (AA) Arabidopsis seeds detected by RT-PCR. cDNAs from NA and AA seeds with different percentages of germination as well as unaged control seeds (100% germination) were used. Different genes in two groups were analyzed. The number of PCR cycles varied depending on their mRNA abundance in the unaged seeds. PCR products were run in agarose gels. (**a**) Analysis of 19 genes in group C (for list of genes, see Table S2). (**b**) Analysis of 15 genes in group D. The germination percentage of a seed sample is indicated above the DNA lane. The gene code is indicated at the left and the length of cDNA amplified by PCR indicated at the right of each row.

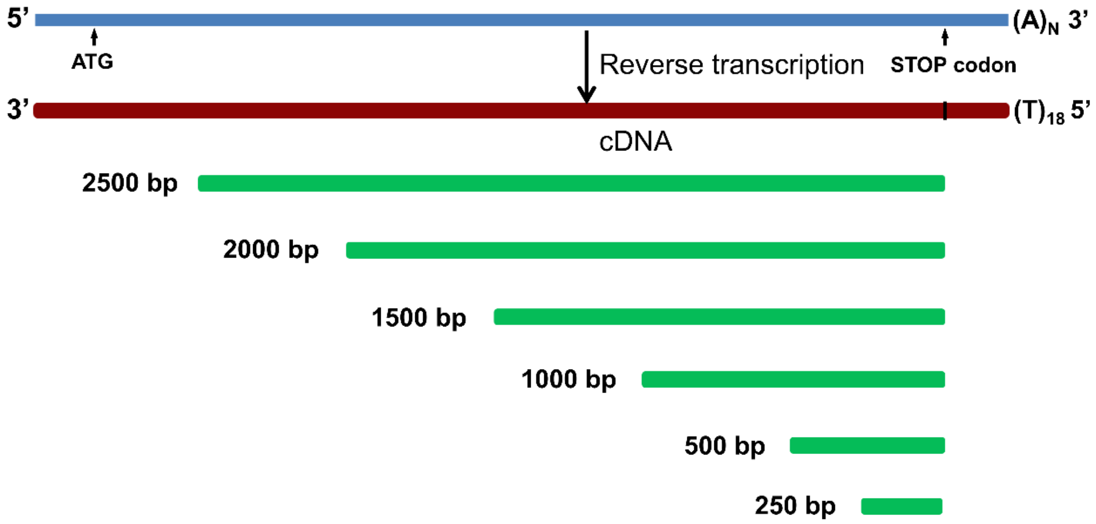

Figure S3. Drawing to show the positions of different fragments on the cDNA derived from a stored mRNA. First-strand cDNAs are synthesized from stored mRNAs with an oligo(dT)$_{18}$ primer. Fragments of different lengths start from the STOP codon and extend towards the 5'-direction of a stored mRNA.

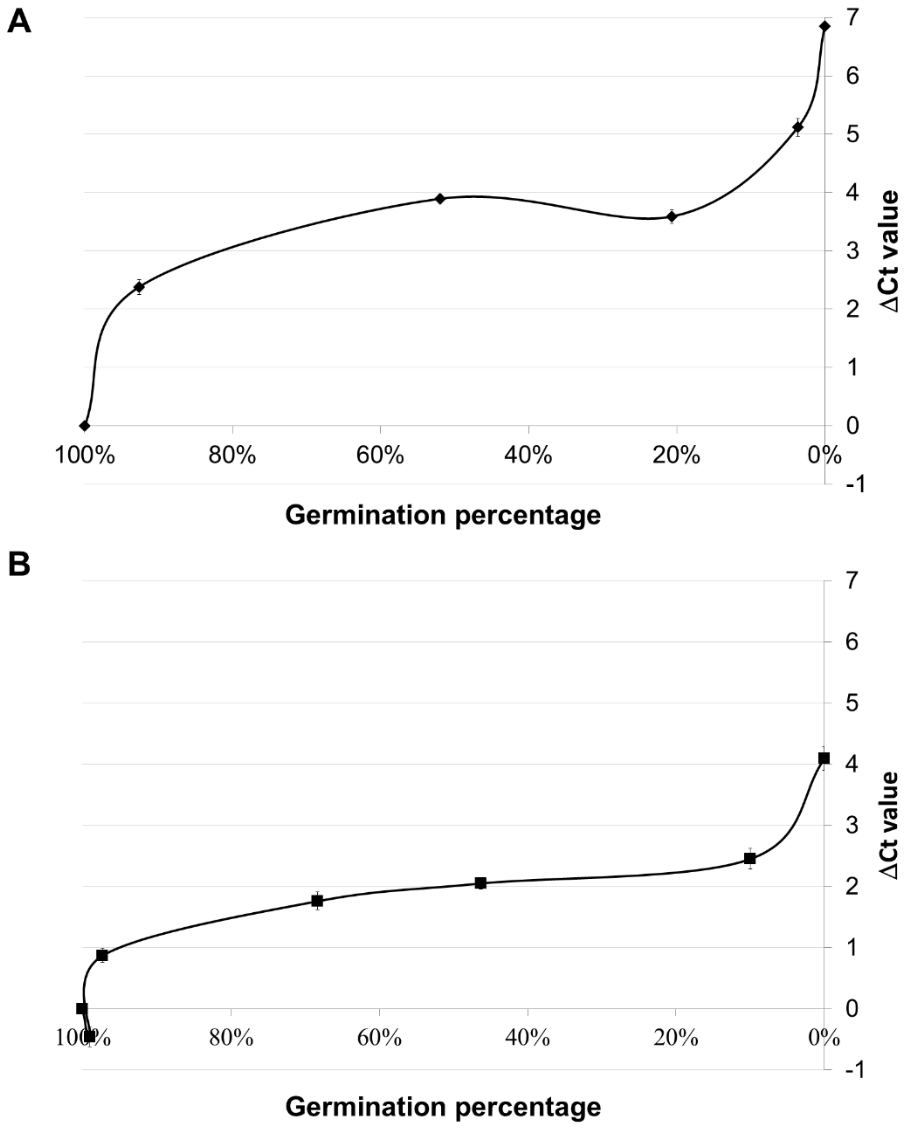

Figure S4. Relationship between △Ct for the cDNA (2500bp fragment) of gene B16 and Arabidopsis seed germination percentage. cDNAs from naturally aged (NA) and artificially aged (AA) seed samples with different percentages of germination were used. **(a)** Relationship between the ΔCt value and germination percentage of six AA samples (with the germination percentage being: 100%, 92.7%, 52%, 20.7%, 3.7% and 0%). **(b)** Relationship between the ΔCt value and germination percentage of five NA samples (with the germination percentage being: 100%, 99%, 97.3%, 68.3%, 46.3% 10% and 0%).

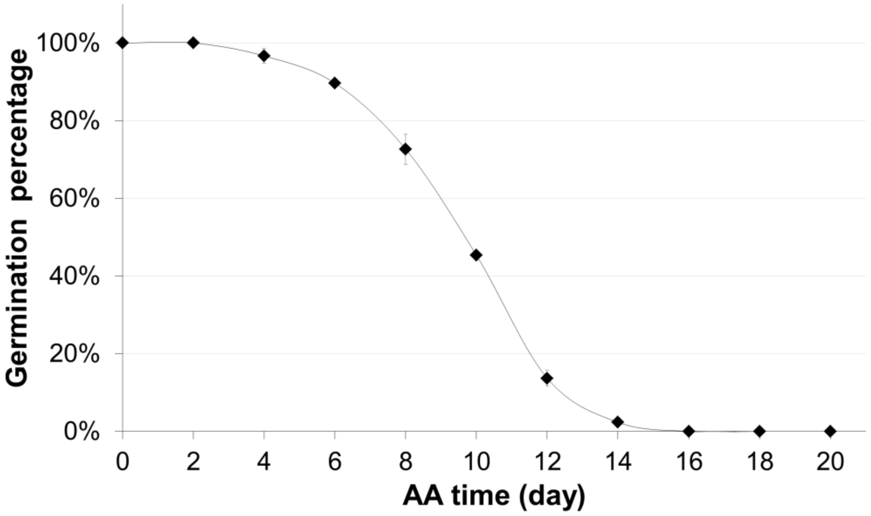

Figure S5. Change in the percentage of germination for Arabidopsis seeds following the artificial aging treatment. Unaged seeds were treated with artificial aging conditions for the indicated times. They were sterilized and then plated on ½ MS plates. The percentage of seeds germinated was obtained after 7 days of incubation.
